# CD38‐Targeting Peptide Vaccine Ameliorates Aging‐Associated Phenotypes in Mice

**DOI:** 10.1111/acel.70147

**Published:** 2025-06-25

**Authors:** Shangcheng Yu, Zhiqiang Li, Yuxiang Tang, Yuling Chen, Yingying Ma, Kunlin Du, Zhaoyun Zong, Kangze Feng, Yali Wei, Limeng Chen, Haiteng Deng

**Affiliations:** ^1^ MOE Key Laboratory of Bioinformatics, Center for Synthetic and Systematic Biology, State Key Laboratory of Complex, Severe, and Rare Diseases Tsinghua University Beijing China; ^2^ Department of Clinical Laboratory Shandong Provincial Hospital Affiliated to Shandong First Medical University Jinan China; ^3^ Department of Nephrology Peking Union Medical College Hospital Beijing China; ^4^ Zhejiang Key Laboratory of Multiomics and Molecular Enzymology Yangtze Delta Region Institute of Tsinghua University Jiaxing China

**Keywords:** aging, CD38, peptide, vaccine

## Abstract

Antiaging vaccines have recently been found to elicit long‐term benefits in slowing the aging process. Meanwhile, high CD38 expression in organs is an aging characteristic contributing to a decreased NAD^+^/NADH ratio. Thus, in the current study, we systematically investigate the effects of a CD38‐targeting peptide vaccine (CD38‐vaccine) on aging‐associated phenotypes in mice. The CD38‐vaccine induces a robust T‐cell immune response, selectively depletes CD38^+^ myeloid cells in the spleen, and ameliorates age‐related physical and cognitive function decline. Metabolically, vaccination improves glucose tolerance, enhances oxygen consumption, and decreases the number of senescent cells and mRNA levels of senescence‐related genes in liver tissues. Vaccination also increases the NAD^+^/NADH ratio in the liver tissues, enhances oxidative metabolism, and reduces glycolysis. These findings indicate that targeting CD38 via vaccination is a promising strategy for ameliorating aging‐associated phenotypes.

## Graphical abstract

Shangcheng Yu et al. revealed that CD38‐targeting peptide vaccine ameliorated aging‐associated physical and cognitive phenotypes in mice. CD38‐vaccine selectively depletes CD38^+^ myeloid cells, improves glucose tolerance, and enhances oxygen consumption. Vaccination also increases the NAD^+^/NADH ratio and decreases the number of senescent cells and mRNA levels of senescence‐related genes in liver tissues.

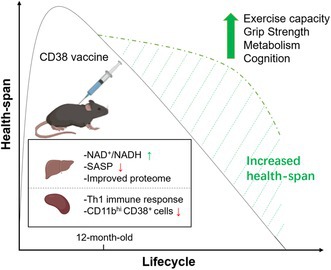

**Abbreviations**
BSA: bovine serum albumin
CD38: cluster of differentiation 38
ELISA: enzyme‐linked immunosorbent assay
ELISPOT: enzyme‐linked immunospot assay
IFN‐γ: interferon‐γ
IL‐4: interleukin‐4
IL‐6: interleukin‐6
IPGTT: intraperitoneal glucose tolerance test
ITT: insulin tolerance test
KLH: keyhole limpet hemocyanin
KSCN: potassium thiocyanate
LC–MS: liquid chromatography coupled to mass spectrometry
MHC: major histocompatibility complex
NAD^+^
: nicotinamide adenine dinucleotide
NAS: nonalcoholic fatty liver disease activity score
SASP: senescence associated secretory phenotype

## 1 Introduction

Aging is a major cause of chronic diseases that negatively impact life expectancy (Campisi et al. 2019). Recent antiaging strategies, including metformin and dietary NAD^+^ precursor supplements, largely based require daily intake (Guarente et al. 2024). Therefore, the development of long‐lasting antiaging approaches is required. Vaccination, with the inherent benefits of requiring few doses and eliciting longer efficacy through autologous immune responses, has demonstrated protective effects against HFD‐induced aging and age‐related metabolic diseases (Yoshida et al. 2020, 2019; Pang et al. 2014; Fukami et al. 2021, 2023; Suda et al. 2021). Previous antiaging vaccines, including CD153 and GPNMB vaccines, have focused primarily on targeting senescent T cells and vascular endothelial cells (Yoshida et al. 2020; Suda et al. 2021). However, other vaccines targeting aging‐related diseases, including ANGPTL3 and Sema3E vaccines, also ameliorate dyslipidemia and glucose intolerance (Yoshida et al. 2019; Fukami et al. 2021). Although a limited number of targets for aging and age‐related diseases have been established for vaccine design (Wu et al. 2024), CD38 has received considerable research attention.

CD38 is predominantly (70%) membrane‐bound and is abundantly expressed in bone marrow and lymphoid tissues. Small molecules targeting CD38 can alleviate glucose intolerance, physical dysfunction, and neuroinflammation (Roboon et al. 2021; Tarrago et al. 2018). More recently, CD38 antibodies have demonstrated efficacy against multiple age‐related syndromes, including fibrosis, systemic sclerosis, NAD^+^ deficiency, and cardiotoxicity (Peclat et al. 2024; Shi et al. 2023; Ugamraj et al. 2022). Indeed, CD38 directs age‐related NAD^+^ decline and mitochondrial dysfunction (Camacho‐Pereira et al. 2016). CD38 also accumulates in the CD11b^+^ macrophages of aged mice (Chini et al. 2020; Covarrubias et al. 2020). Meanwhile, high CD38 expression causes cells to undergo an epithelial–mesenchymal transition process, suggesting that CD38 is an aging target (Wang, Hu, Wang, et al. 2018; Wang, Hu, Yang, et al. 2018). However, it remains unclear whether the selective depletion of CD38^+^ cells by vaccination can attenuate age‐related physiological performance and pathological changes.

In this study, we investigate the effects of a CD38‐targeting peptide vaccine (CD38‐vaccine) on aging‐associated phenotypes in mice. We identified a novel CD38 peptide (^2^ANYEFSQV^9^) with high immunogenicity. Targeting CD38 via prime‐boost vaccination in C57BL/6J mice positively affected various aspects of health and physiology long‐term. Immunological and proteomic analyses elucidated the potential mechanisms underlying the protective effect. Collectively, this study provides insights for investigating vaccine‐based anti‐aging approaches targeting CD38.

## 2 Results

### 2.1 Selection of Antigen Sequences for a CD38 Peptide Vaccine

**FIGURE 1:**
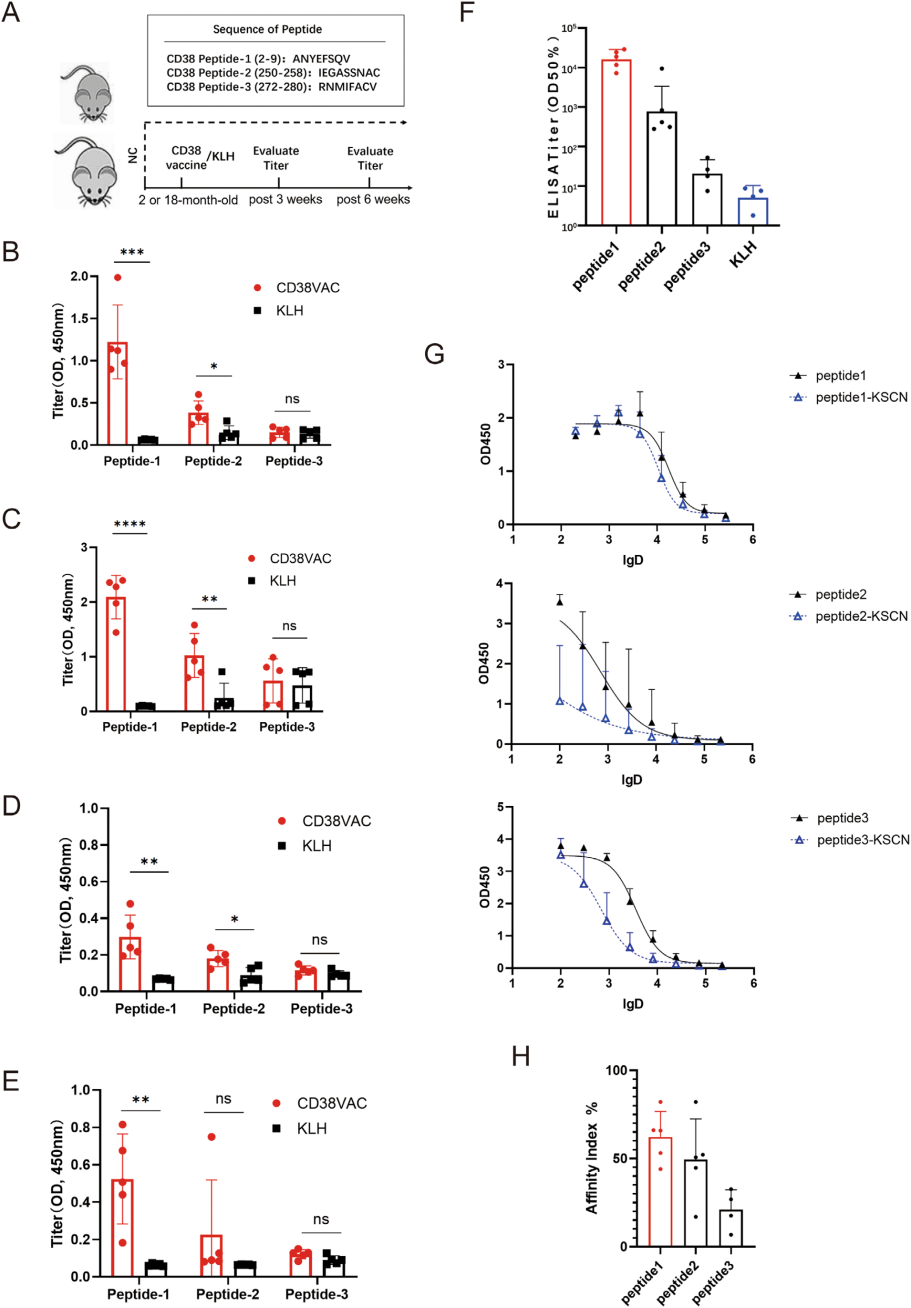
Development of a CD38 vaccine. (A) Schematic diagram of vaccine immunization‐serological test protocol and sequence of CD38 epitope candidates (peptide‐1, −2, −3). (B–E) Analyses of young mice (2‐month‐old) (B, D) and aged mice (18‐month‐old) (C, E) serum 3 weeks (B, C) and 6 weeks (D, E) after KLH (KLH) or the KLH‐conjugated‐CD38‐peptide‐1 (CD38vac) vaccine. Serum dilution at 4050‐fold were measured by ELISA (*n* = 5/group, each dot represents an individual mouse). (F) Analyses of young mice (2‐month‐old) serum 6 weeks post‐immunization. Antibody titer against indicated peptide‐BSA following immunization of indicated peptides conjugated with KLH as assessed by ELISA (*n* = 4–5/group, each dot represents an individual mouse). Values are reported as the serum dilution giving half‐maximal binding (OD 50%). (G) CD38‐peptide specific antibody binding curve challenged with or without KSCN (*n* = 4–5/group; serum dilution from 1:100 to 1:270,000; lgD: Log10[dilution factor]). (H) Avidity index of CD38 peptide candidate vaccine, defined as ratios of serum dilution giving half‐maximal binding (OD 50%) with or without KSCN challenge (*n* = 4–5/group, paired for ratio calculation). All data were analyzed by the two‐tailed Student's *t*‐test (B–E); Values represent the mean ± SD; *p < 0.05, **p < 0.01, ***p < 0.001, ****p < 0.0001, ns = not significant.

To select antigen sequences with high immunogenicity for CD38 peptide vaccine development, three epitopes were selected as candidate antigens: peptide‐1 (amino acids (aa) 2–9), peptide‐2 (aa 250–258), and peptide‐3 (aa 272–280). Each peptide was conjugated to KLH and mixed with an alum adjuvant to establish CD38 vaccines. After 3 and 6 weeks of vaccination, blood was collected from control and vaccinated mice, and the effects of the candidate antigens on epitope‐specific antibody production were evaluated (Figure 1A). After 3 and 6 weeks of immunizing 2‐month‐old mice, the specific antibody levels against CD38 peptides were significantly higher in mice immunized with KLH‐conjugated CD38 peptide‐1 or ‐2. However, robust antibody responses were not observed in mice immunized with KLH‐conjugated CD38 peptide‐3 (Figure 1B,C). To further assess the immunogenicity of the candidates, 18‐month‐old mice were immunized and specific antibody levels were measured at 3 and 6 weeks post‐vaccination. The specific antibody levels against CD38 peptides were significantly higher in mice immunized with KLH‐conjugated CD38 peptide‐1, while a robust antibody response was not observed in mice immunized with KLH‐conjugated CD38 peptide‐2 3 or 6 weeks after the first immunization (Figure 1D,E). Evaluation of antibody production by ELISA also showed that the titers against mouse CD38 peptide‐BSA 6 weeks after the first immunization was more robust in the peptide‐1 group than in the other groups (Figure 1F). Moreover, KSCN‐ELISA was employed to evaluate the avidity of candidate‐induced specific antibodies against CD38 epitopes (Portilho et al. 2023). Here, serum‐specific IgG titers were measured 6 weeks after immunization with or without the chaotropic agent KSCN (Figure 1G). The avidity index of the CD38 peptide‐1 vaccine‐induced antibodies exceeded 60%, whereas the others did not (Figure 1H). These results demonstrated that the “ANYEFSQV” sequence was highly immunogenic; the CD38 peptide‐1 vaccine‐induced antibodies effectively recognized this epitope. Therefore, it was selected as an antigen for further CD38‐vaccine studies.

### 2.2 Cellular Immune Responses in Aged Mice Immunized With the CD38 Vaccine

**FIGURE 2:**
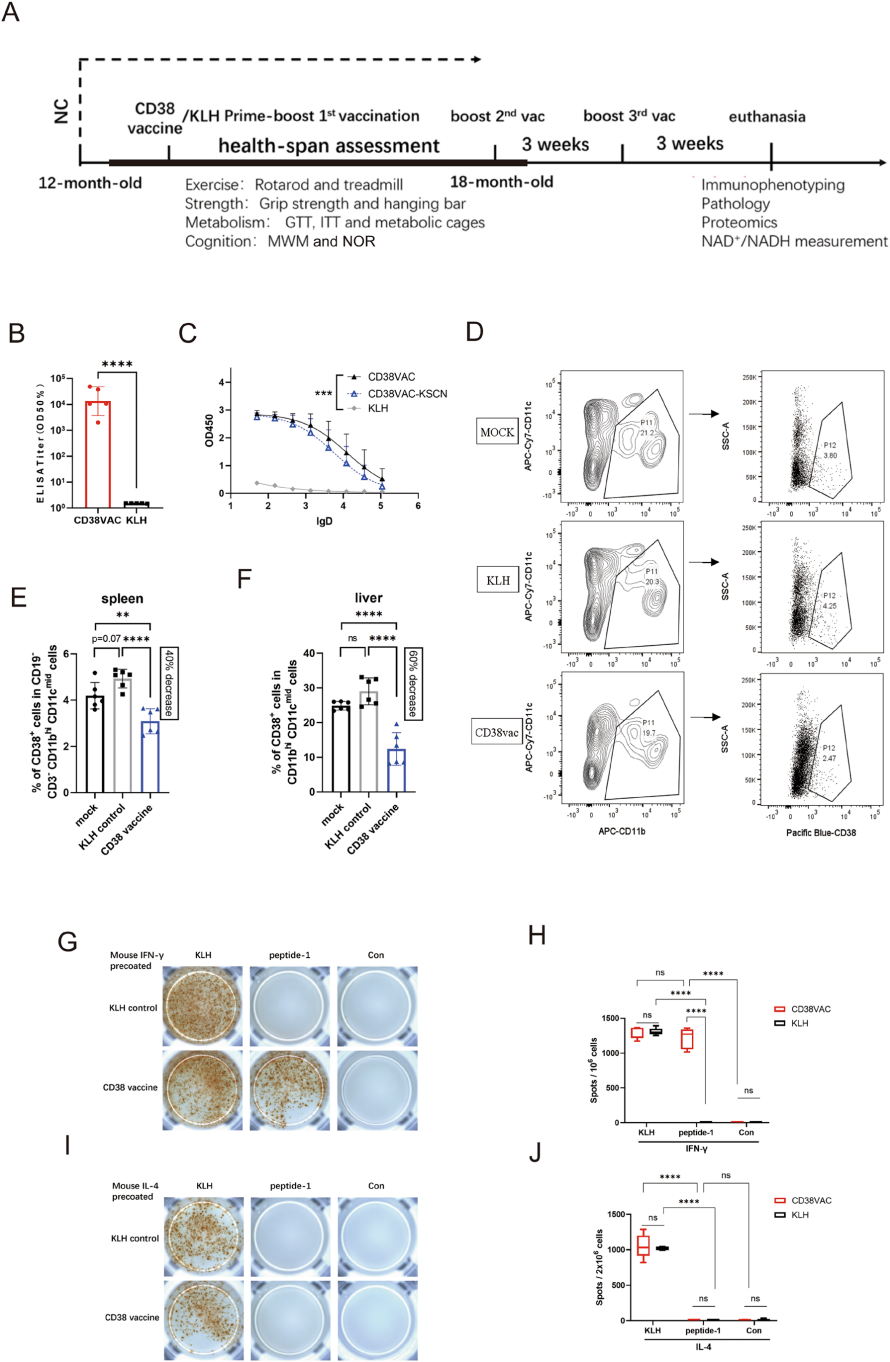
Characterization of immune response to CD38 vaccine. (A) Schematic diagrams of the prime‐boost vaccination strategy and healthspan assessment. (B) Antibody titer against CD38 peptide‐BSA at 20 months old with KLH or CD38 vaccine as assessed by ELISA (*n* = 5/group, each dot represents an individual mouse). Values are reported as the serum dilution giving half‐maximal binding (OD 50%). (C) CD38‐peptide specific antibody binding curve challenged with or without KSCN (*n* = 5/group; serum dilution from 1:50 to 1:109,350; lgD: log10[dilution factor]) (D–E) Proportions of CD38+ cells in CD19‐ CD3‐ CD11bhi CD11cmid cells (P12 in Gate P11) in splenic tissues of male C57BL/6J mice (*n* = 6/group) at 20 months old with or without KLH or CD38 vaccine; (F) Proportions of CD38+ cells in CD11bhi CD11cmid cells in liver tissues of male C57BL/6J mice (*n* = 6/group) at 20 months old with or without KLH or CD38 vaccine (G–J) ELISPOT assay of IFN‐γ (G) or IL‐4 (I) produced by splenocytes from male C57BL/6J mice (*n* = 5/group) at 20 months old administered KLH (KLH) or CD38 vaccine (CD38vac). Splenocytes were stimulated with KLH, CD38 peptide‐1 (CD38p), or PBS (Con). Right panels indicate the number of spots per 106 cells (H), or 2 × 106 cells (J) in the indicated groups. No outliers or abnormal values were excluded by boxplot analysis. All data were analyzed by two‐tailed Student’s *t*‐test (B) and ANOVA followed by Sidak's multiple comparisons test (C, E, F), or by Tukey’s multiple comparison test (H, J); values represent the mean ± SD; **p < 0.01, ***p < 0.001, ****p < 0.0001, ns = not significant.

We used a prime‐boost vaccination strategy to explore the effects and mechanisms of the CD38 vaccine. In the CD38 vaccine group, C57BL/6J mice were fed a normal diet and subcutaneously immunized with the CD38 peptide vaccine (CD38‐vaccine mice) at 12 months old, followed by healthspan assessments between 15 and 18 months old. At 18 months and 3 weeks of age, the mice were immunized with the CD38 peptide vaccine by subcutaneous injection (Figure 2A). Mice in the control group (KLH mice) were simultaneously immunized with KLH. At 20 months old, both groups were euthanized. Tissue and blood samples were collected for immunophenotyping, pathology, proteomics, and other experiments.

We first examined the immune response for 20‐month‐old mice. Serum IgG titers were measured, showing that a robust antibody response was presented in CD38‐vaccine mice while it was not detected in KLH mice (Figure 2B and Figure S2A). The antibody avidity index in CD38‐vaccine mice at 20 months is similar to that in young mice at 3 and 6 weeks post CD38 peptide‐1 vaccination (Figure 2C and Figure S2B). For immunophenotyping, the proportional distribution of immune cells was assessed in the spleen of CD38‐vaccine mice and KLH mice. Considering that CD38^+^ myeloid cells (CD11b^hi^ CD11c^mid^) reportedly accumulate and drive age‐associated NAD^+^ decline (Chini et al. 2020; Covarrubias et al. 2020), a multi‐color flow cytometry strategy was employed to simultaneously detect CD4^+^ T cells, CD8^+^ T cells, CD19^+^ B cells, and CD11b^hi^ CD11c^mid^ cell subsets in the spleen and CD3^+^ T cells, CD11b^hi^ CD11c^mid^ cells in the liver (Figure S1). WT C57BL/6J mice of the same age served as a control for the effects of vaccination (mock group).

In CD38‐vaccine mice, the proportion of CD38^+^ cells was significantly lower than that in the KLH mice in the splenic and liver tissues of C57BL/6J mice (*n* = 6/group) at 20 months (Figure S2C,D). The proportion of CD38^+^ cells in CD19^−^CD3^−^CD11b^hi^CD11c^mid^ cells (P12 in Gate P11) in the splenic tissues of C57BL/6J mice (*n* = 6/group) at 20 months of age was slightly increased compared to the mock group and significantly lower (~40% decrease) than that in the KLH mice (Figure 2D,E). The proportion of CD38^+^ cells in CD11b^hi^CD11c^mid^ cells in the liver tissues of C57BL/6J mice (*n* = 6/group) at 20 months was lower (~60% decrease) than that in the KLH mice (Figure S2E and Figure 2F). These results highlight that vaccination specifically targets CD11b^hi^CD11c^mid^ CD38^+^ cells.

Since “ANYEFSQV” is predicted to have a strong binding with H2‐Kb among mouse CD38 peptides (11.4 nM) (Table S2), we propose that the antigen‐specific cellular immune response also contributed to CD38‐vaccine mice. Additionally, the mRNA expression levels of Cd8a, Ifng, and Gzmb were increased in the splenocytes of CD38‐vaccine mice, while Cd38 was decreased (Figure S2F). To further investigate the potential cellular immune response to the CD38 vaccine, an ELISpot test was performed on mouse splenocytes to characterize T cell reactivity. KLH was used as the positive control to stimulate splenocytes (Pang et al. 2014). In CD38 vaccine mice, stimulating splenocytes with KLH led to an increase in IL‐4 and IFN‐γ production, while stimulating splenocytes with the CD38 peptide‐1 elicited a similar increase in IFN‐γ production (Figure 2G,H), without impacting IL‐4 (Figure 2I,J). In KLH mice, stimulating splenocytes with KLH increased IL‐4 and IFN‐γ production, while stimulating splenocytes with CD38 peptide‐1 did not affect IL‐4 or IFN‐γ production. These results indicated that the CD38 vaccine elicited an antigen‐specific immune response; however, it primarily activated Th1 immunity, not Th2. Taken together, these results suggest that the CD38 vaccine decreases the proportion of CD11b^hi^ CD11c^mid^ CD38^+^ cells and induces a Th1‐mediated antigen‐specific cellular immune response.

### 2.3 CD38 Peptide Vaccine Prevents Physical and Cognitive Decline in Aged Mice

Next, the effect of CD38 peptide vaccination on aging‐associated phenotypes, specifically physical and cognitive dysfunction, was evaluated. A previously described healthspan assessment protocol, including physical activity, grip strength, cognitive function, and metabolic status, was performed from 15 to 20 months of age (Sukoff Rizzo et al. 2018; Bellantuono et al. 2020).

**FIGURE 3:**
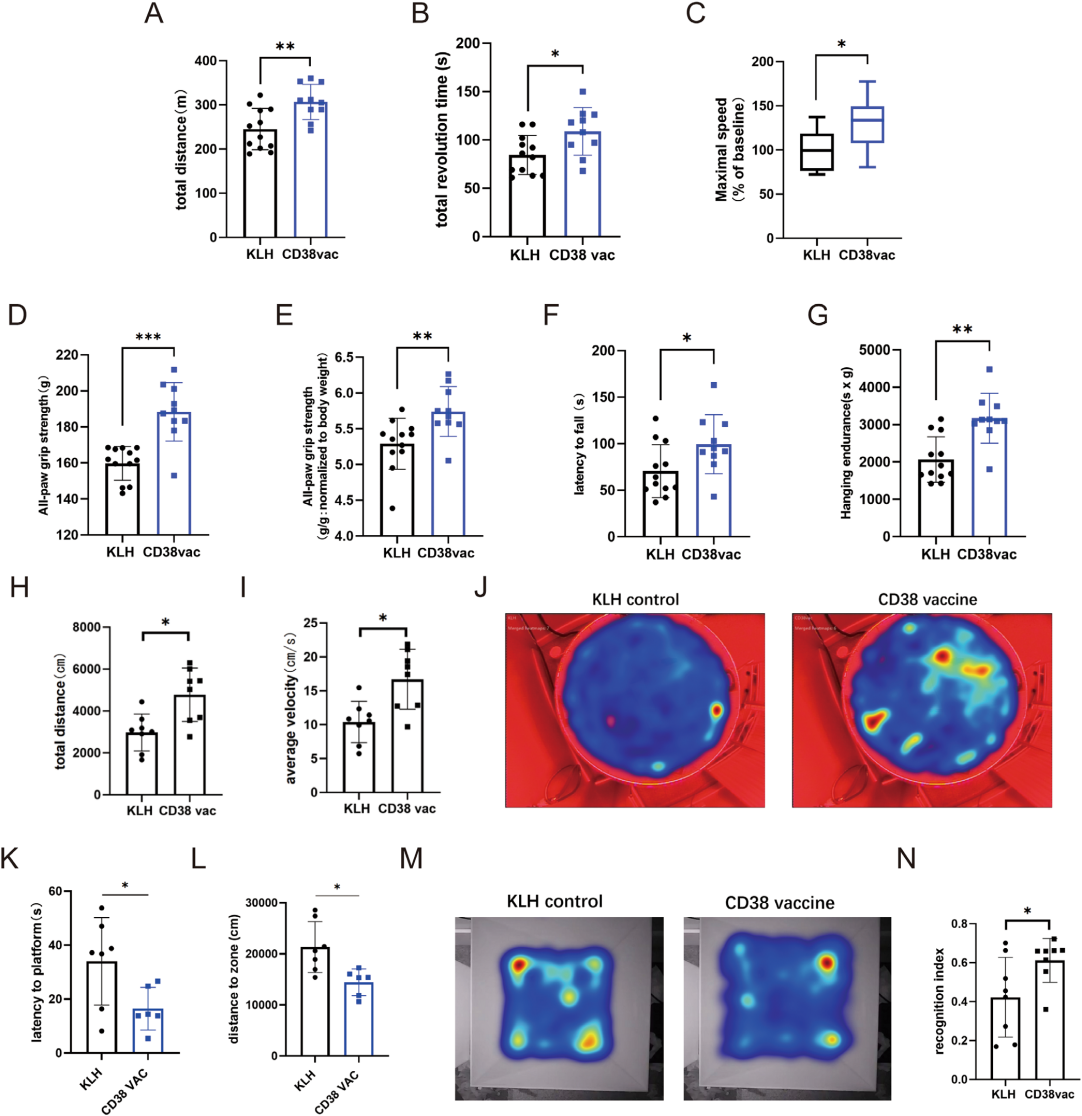
CD38 vaccine prevents physical and cognitive decline in naturally‐aged mice. (A–G) Total walking distance on treadmill (A), total revolution time and maximal walking speed (relative to baseline) on rotarod (B, C), all‐paw grip strength (D, E) and hanging endurance (F, G) of KLH mice and CD38‐vaccine mice at 15–16 months old (male and female, *n* = 12, 10; details for sex separated data shown in Figure S3) (H, I) Total movement distance (H) and average exploring velocity (I) of KLH mice (*n* = 8) and CD38 vaccine mice (*n* = 8) at 20 months old in open field test model. (J–N) CD38 peptide vaccine prevents cognitive decline in male C57BL/6J mice. Accumulative swimming trajectory heatmaps (J), latency to platform (K), accumulative distance to platform zone (L) were recorded in Morris water maze model (*n* = 7, 6; at 16 months old). Accumulative trajectory heatmaps (M) and recognition index (N) were recorded in novel object recognition model (*n* = 8, 8; at 20 months old). All data were analyzed by an unpaired two‐tailed Student's *t*‐test. Data are shown as individual dot plots and box‐and‐whisker plots, where a box extends from the 25th to 75th percentile with the median shown as a line in the middle, and whiskers indicate the smallest and largest values; values represent the mean ± SD; *p < 0.05, **p < 0.01, ***p < 0.001, ns = not significant.

First, the effects of the CD38 vaccine on preventing physical decline were assessed. Vaccination with the CD38 peptide at 12 months old prevented a decline in total walking distance (via treadmill; Figure 3A), total revolutions, maximal walking speed (relative to baseline) (via rotarod; Figure 3B–C), all‐paw grip strength (via grip meter; Figure 3D,E), and hanging endurance (via hanging test; Figure 3F–G) at 15 and 16 months. Effects of vaccination were equally applied into both male and female mice (Figure S3B–E). CD38 peptide vaccine also enhanced rotarod performance and grip‐strength in an age‐dependent manner (Figure S3F,G). Similarly, the total movement distance and average exploring velocity of CD38 vaccine mice at 20 months were higher than those of KLH mice in the 5‐min Open Field Test (Figure 3H,I).

To investigate whether the CD38 vaccine prevents cognitive decline in aged mice, a Morris Water Maze experiment was designed, and the latency to the platform on the final day of the hidden platform period was measured at 16monthsold. Swimming routes and velocity were recorded during the 2‐min free exploration period (Figure S3J–K). The movement heatmaps demonstrated that CD38 vaccine mice were likelier to shuttle through the platform zone (Figure 3J). The latency to the platform of CD38 vaccine mice was shorter than that of KLH mice (Figure 3K). The cumulative distance to the platform zone during the 2‐min free exploration period of CD38 vaccine mice was shorter than that of KLH mice (Figure 3L). Next, we used the novel object recognition model to assess cognitive function at 20 months. Likewise, CD38‐vaccine mice spent more time to explore the novel object (upper right, area 2) compared with KLH mice (Figure 3M,N). The appearance of CD38‐vaccine mice looked younger compared to KLH mice at 20 months (Figure S3A). To quantitatively assess the level of frailty, we calculated the cumulative frailty score (including 27 parameters) in mice. Statistically, both male and female CD38‐vaccine mice exhibited improved frailty scores at 18 and 20 months (Figure S3L,M). These data consistently supported the prominent capacity of the CD38 vaccine preventing physical and cognitive decline during the early aging process.

### 2.4 CD38 Peptide Vaccine Ameliorates Metabolic Dysfunction‐Associated Features in Aged Mice

**FIGURE 4:**
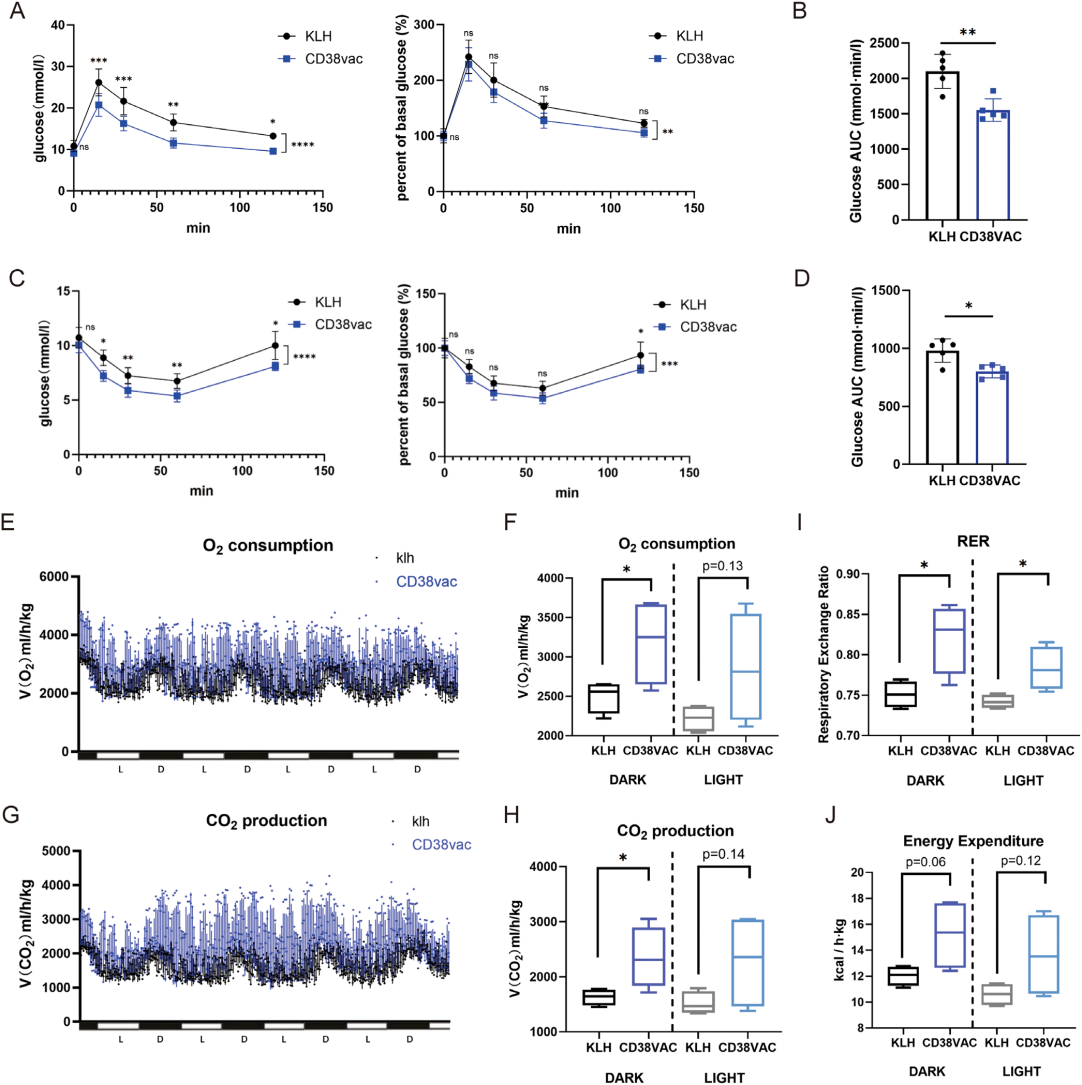
CD38 peptide vaccine ameliorates metabolic dysfunction‐associated features in naturally‐aged mice. (A) Blood glucose concentrations and percent basal glucose in mice immunized with KLH or CD38‐vaccine (*n* = 5/group) as determined by the intraperitoneal glucose tolerance test (ipGTT) at 17 months old. (B) Area under the curve (AUC) of blood glucose levels as determined by the ipGTT using the trapezoidal rule. (C) Blood glucose concentrations and percent basal glucose in mice immunized with KLH or CD38‐vaccine (*n* = 5/group) as determined by the insulin tolerance test (ITT) at 17 months old. (D) AUC of blood glucose levels, as determined by the ITT, estimated using the trapezoidal rule. (E–J) CD38 peptide vaccine enhanced O_2_ consumption (E, F), CO_2_ production (G, H), RER (I), and energy expenditure (J) of mice during the awake period measured with metabolic cages at 17 months old. L: Light, D: Dark, *n* = 4/group. All data were analyzed by an unpaired two‐tailed Student's *t*‐test (B, D, F, H, I, J) or Tukey's multiple comparison test (A, C). Data are shown as individual dot plots and box‐and‐whisker plots, where a box extends from the 25th to 75th percentile with the median shown as a line in the middle, and whiskers indicate the smallest and largest values; Values represent the mean ± SD; *p < 0.05, **p < 0.01, ***p < 0.001, ****p < 0.0001, ns = not significant.

Metabolic health, especially glucose homeostasis and energy balance, is a key factor for evaluating the efficacy of geroprotective therapeutics. With aging, mice exhibit features associated with metabolic dysfunction, including glucose intolerance and lower respiratory metabolic rates (Amorim et al. 2022). Hence, the ability of the CD38 peptide vaccine to ameliorate metabolic dysfunction‐associated features in a naturally aged mouse model was evaluated. As determined by the ipGTT and ITT at 17 months of age, glucose tolerance (Figure 4A,B) and insulin sensitivity (Figure 4C,D) were improved in CD38 vaccine mice compared to KLH mice. Hence, the vaccine improved systemic metabolic health.

Next, the effect of the CD38 vaccine on energy balance was evaluated. Individual O_2_ consumption (Figure 4E) and CO_2_ production (Figure 4G) were recorded in the metabolic cages. The CD38 peptide vaccine enhanced O_2_ consumption (Figure 4F) and CO_2_ production (Figure 4H) in mice, especially during the awake period (dark), indicating enhanced oxidative phosphorylation (OXPHOS) in vivo. We found that the respiratory exchange ratio (RER) and energy expenditure (EE) were increased in CD38‐vaccine mice (Figure 4I,J). The CD38 peptide vaccine enhanced food and drink intake during metabolic cage monitoring (Figure S4A,B). Locomotor activities were slightly increased in CD38‐vaccine mice at 17 months (Figure S4C). Since the average weight of CD38‐vaccine mice was lower than that of KLH mice (Figure S4D), an analysis of covariance (ANCOVA) test was used to exclude the confounding effect of body weight on basal heat production (Tschöp et al. 2012). Taken together, energy expenditure, oxygen consumption, and RER were indeed increased in CD38‐vaccine mice, irrelevant to body weight (Figure S4E,F). These results suggest that the CD38 vaccine ameliorates metabolic dysfunction‐associated features during the early aging process.

### 2.5 CD38 Peptide Vaccine Reduces Cellular Senescence and Maintains NAD ^+^/NADH Levels

**FIGURE 5:**
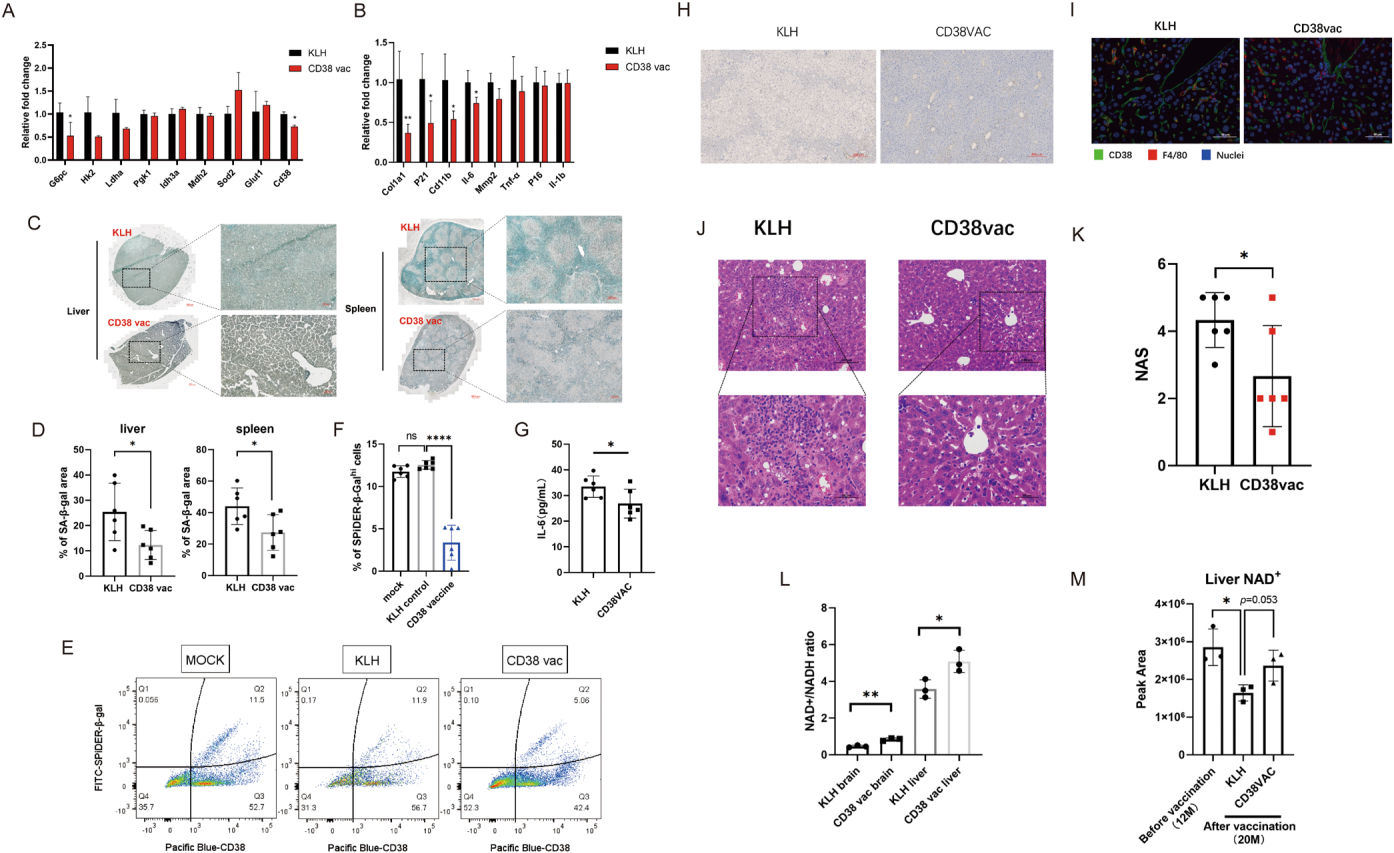
Decreased senescent cells and elevated NAD^+^ in tissues of naturally‐aged mice following CD38‐peptide vaccination. (A) Liver mRNA levels of glucose metabolism‐related genes (*n* = 3–5 mice/group) at the end of the treatment. (B) Liver mRNA levels of cellular senescence‐related genes (*n* = 3–5 mice/group) at the end of the treatment. (C) SA‐β‐gal assay of liver and spleen tissue obtained from KLH mice or CD38 vaccine mice (20‐month‐old). (D) Quantification of SA‐β‐gal activity across tissues. (*n* = 6/group) (E‐F) The proportions of CD38^+^ and SPiDER‐β‐gal^+^ cells in liver tissues of male C57BL/6J mice (*n* = 6/group) at 20 months old with or without KLH or CD38 vaccine. Senescent cells were defined as SPiDER‐β‐gal^+^ cells (Q1 + Q2). (G) The ELISA results of IL‐6 concentrations in serum obtained from KLH mice or CD38 vaccine mice (*n* = 6/group, 20‐month‐old). (H) Immunohistochemical staining of mouse CD38 in liver tissue obtained from KLH mice and CD38 vaccine mice. Scale bar, 200 μm. (I) Immunofluorescence staining of CD38 (green), macrophage marker antigen F4/80 (red) and nuclei with DAPI (blue) in liver from KLH mice or CD38 vaccine mice (20‐month‐old), representative of *n* = 3/group. Scale bar: 50 μm. (J) Representative images of hematoxylin and eosin (H&E)‐stained liver sections. Scale bars, 100 μm (up) and 50 μm (down). (K) Nonalcoholic fatty liver disease activity score (NAS), as estimated based on H&E‐stained sections (*n* = 6/group). (L) Relative NAD^+^/NADH ratio of liver and brain tissue obtained from male KLH mice and CD38 vaccine mice (*n* = 3 mice/group, 20‐month‐old). (M) LC–MS quantification of NAD^+^ in the liver from mice before vaccination (12 months old), KLH mice (20 months old), and CD38‐vaccine mice (20 months old) (*n* = 3 mice/group). All data were analyzed by two‐tailed Student's t‐test (A, B, D, G, K, L) and ANOVA followed by Sidak's multiple comparisons test (F, M); values represent the mean ± SD; *p < 0.05, **p < 0.01, ****p < 0.0001, ns = not significant.

Aging is associated with various molecular and histological tissue changes, which coincide with increased cellular senescence (Baker et al. 2016). Given the prominent efficacy of the CD38 vaccine in preventing physical, cognitive, and metabolic functional decline, the underlying mechanisms were investigated. The mRNA expression levels of glucose metabolism‐related genes were evaluated in the liver tissues. The CD38 vaccine decreased the mRNA expression of multiple key glycolytic enzyme genes, including *G6pc*, *Hk2*, and *Ldha* (Figure 5A). Cell cycle arrest in senescent cells is primarily mediated through the P53–CDKN1A (P21) pathway and the retinoblastoma (RB)–CDKN2A (P16) pathway (Sheekey and Narita 2023; Martínez‐Zamudio et al. 2017). In the liver tissue, the mRNA levels of *P21* were markedly decreased, while *P16* was unaffected by the CD38 vaccine (Figure 5B). A decrease in p21 protein expression was also observed in liver and spleen tissues of CD38‐vaccine mice (Figure S5A).

Next, the effects elicited by the CD38 vaccine on senescence‐associated secretory phenotype (SASP) factors in mouse liver tissue were evaluated. In liver tissue, the mRNA levels of *Col1a1*, *Mmp2*, and *Il6* were markedly decreased in CD38 vaccine mice (Figure 5B). IL‐6 levels in serum of CD38‐vaccine mice were also lower than those in KLH mice (Figure 5G). In addition, the mRNA expression of *Cd11b*, one of the myeloid‐marker gene, was also decreased in CD38 vaccine mice, consistent with the cell population changes observed by flow cytometry (Figure S2G,H). Moreover, in liver and spleen tissues, the β‐gal activity was decreased in CD38‐vaccine mice compared with KLH mice (Figure 5C,D). To quantitatively assess whether CD38 peptide vaccine reduces cellular senescence in the liver, flowcytometry with anti‐CD38 Ab and SPiDER‐β‐gal probe were applied into analysis of 20‐month‐old mice (Figure 5E). In accordance with SA‐β‐gal staining, senescent cells in CD38 vaccine mice were reduced (Figure 5F). About 20% of the CD38^+^ cells had high SA‐beta‐gal activity in control group, while only 5% of the CD38^+^ cells had high SA‐beta‐gal activity in CD38‐vaccine mice (Figure S5B). These results showed that the CD38‐vaccine decreased cellular senescence in solid organs.

To examine whether the CD38 vaccine directly targeted CD38, the expression of CD38 mRNA and protein was assessed in liver tissues. The CD38 peptide vaccine reduced CD38 expression at the mRNA and protein levels in the liver tissues (Figure 5A,H), consistent with the decreased CD38^+^ cells observed by flow cytometry previously (Figure S2D). Previous studies have shown that CD38‐expressing macrophages caused age‐related NAD^+^ decline (Chini et al. 2020; Covarrubias et al. 2020; Wu and Zhang 2020). Considering the reduction of CD11b subsets in CD38‐vaccine mice, we hypothesized that vaccination caused fewer CD38‐expressing macrophages localized in the liver. IF staining for F4/80+ Kupffer cells (red) and CD38 (green) revealed the decreased number of macrophages in which CD38 and F4/80 were co‐expressed in liver sinusoids of CD38‐vaccine mice (Figure 5I). The number of CD38^+^ and CD11b^+^ cells was decreased in the spleen and liver of CD38‐vaccine mice (Figure S5C). Histological analysis also revealed that steatosis (score 0–3), hepatocyte ballooning (score 0–2), and lobular inflammation (score 0–3), as estimated by the nonalcoholic fatty liver disease activity score (NAS) (Kleiner et al. 2005), were alleviated in CD38 vaccine mice relative to KLH mice (Figure 5J,K). The histological analysis of spleen and kidney tissue sections revealed that there were no significant differences in inflammation or fibrosis between CD38‐vaccine mice and KLH mice (Figure S5D).

Redox homeostasis, as reflected by antioxidant gene expression and NAD^+^/NADH levels in solid tissues, is associated with mitochondrial dysfunction and aging‐related neurodegenerative diseases, including Alzheimer's diseases (Lautrup et al. 2019; Verdin 2015; Rimal et al. 2023; Flynn and Melov 2013). Hence, the mRNA levels of redox homeostasis‐related genes and NAD^+^ levels were evaluated in liver tissues after vaccination. The CD38 vaccine upregulated *Sod2* mRNA expression (Figure 5A) and increased the NAD^+^/NADH ratio in mouse liver and brain tissues at 20 months of age (Figure 5L). CD38‐vaccine also partly restored the age‐related NAD^+^ decline in mouse liver tissues (Figure 5M). Vaccination increased total NAD^+^ levels in mouse spleen and brain tissues at 20 months (Figure S5E,F). Taken together, these results suggest that the CD38 vaccine reduces cellular senescence and maintains NAD^+^/NADH levels.

### 2.6 CD38 Peptide Vaccine Ameliorates Abnormal Metabolic‐Related Proteome Changes in the Liver

**FIGURE 6:**
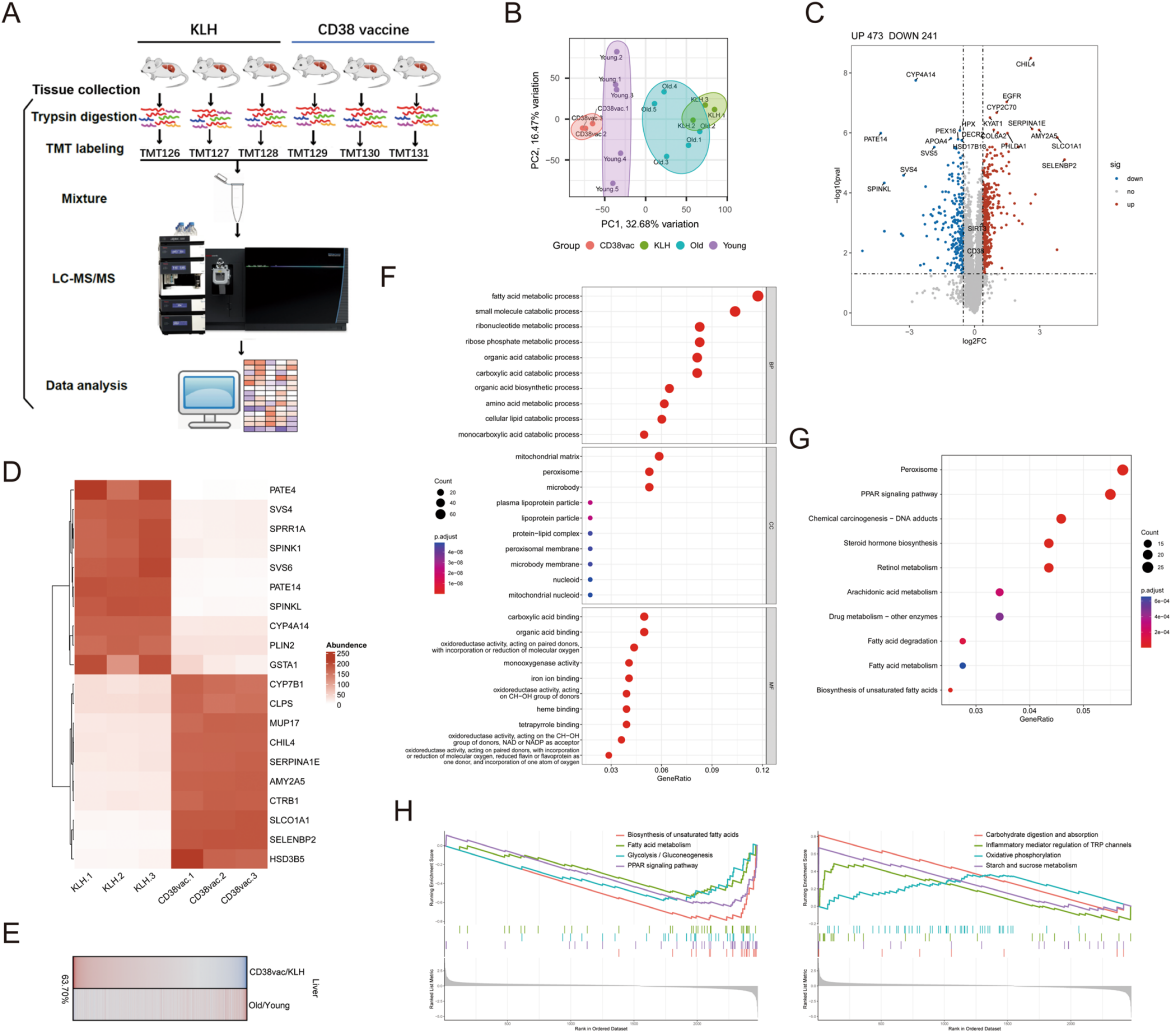
CD38 peptide vaccine restores liver proteome. (A) Schematic diagram of TMT6plex quantitative proteomics sample process from the liver tissue of KLH mice and CD38 vaccine mice at 20 months old. (B) PCA was performed on differentially‐expressed proteins (DEPs) obtained from the liver tissue of young and old KLH and CD38 vaccine mice. Each data point corresponds to the PCA analysis of each subject. (C) Volcano plot of proteomics data from the liver tissue of CD38 vaccine mice versus KLH mice, red dots indicate the proteins with adjusted *p* < 0.05 and fold change > 1.3; blue dots represent proteins with fold change < 0.7 and adjusted *p* < 0.05. (D) Heatmap showing the normalized expression levels of top ten DEPs from the proteomics analysis of mouse liver tissue. (E) Gene expression profile comparing the protein level significantly upregulated (red) and downregulated (blue) by natural aging or CD38‐vaccine in liver tissue. (F–H) Bioinformatics analysis of TMT6plex quantitative proteomics. Gene Ontology (E), including top ten GO‐BP, GO‐CC, and GO‐MF terms enriched by DEPs. (F) Top ten KEGG pathways enriched by DEPs. (G) Gene set enrichment analysis (GSEA) of up‐regulated and down‐regulated pathways.

To investigate the mechanism directly responsible for the improvements in aging‐associated phenotypes in CD38 vaccine mice, the global proteome in the mouse liver was systematically analyzed using TMT6plex quantitative proteomics (Figure 6A). Overall, the proteome‐wide shotgun approach quantified 6950 proteins, providing resources for investigating vaccine‐based antiaging approaches that target CD38. Principal component analysis (PCA) demonstrated a clear effect of the CD38 vaccine, along with a shift in the global gene expression profile toward changes against natural aging (Figure 6B). Volcano plots were created for the proteomics data for the liver tissues of CD38 vaccine mice versus KLH mice (Figure 6C); the abundances of 518 proteins were increased, and 265 proteins were decreased in CD38 vaccine mice (Table S3). The top 10 differentially expressed proteins (DEPs) from the bioinformatics analysis of mouse liver tissue are presented as heat maps (Figure 6D). The expression level of CD38 was downregulated in the liver of CD38‐vaccine mice (Figure S5G). Further analysis of the proteome profile revealed that the majority of the proteins (63.7%) whose expression was modified by natural aging and CD38 peptide vaccination were shifted in the opposite direction in the liver, which supported that CD38 peptide vaccination systematically improved the abnormal aging‐associated proteome (Figure 6E) (Yu et al. 2020).

The liver plays a central role in the maintenance of glucose homeostasis and has been proposed as one of the main targets of multiple antiaging therapeutics, including metformin and NAD^+^ precursors (Radziuk et al. 2003; Mitchell et al. 2018). Thus, to clarify the potential mechanisms by which the CD38 peptide vaccine ameliorates metabolic dysfunction‐associated features and redox imbalance, bioinformatics analysis was employed to identify the functional pathways and biological processes responsible for this phenomenon. Gene Ontology (GO) function annotation and Kyoto Encyclopedia of Genes and Genomes (KEGG) pathway analysis revealed multiple enriched metabolism‐related processes. The GO terms for the upregulated DEPs in the CD38 mouse liver samples under “biological process” were primarily related to fatty acid metabolic processes and catabolism (Figure 6F), under “cellular component” were associated with mitochondrial matrix and peroxisome (Figure 6F), and under “molecular function” were related to organic acid binding and oxidoreductase activity (Figure 6F). KEGG analysis revealed that these DEPs were primarily enriched in the peroxisome and PPAR signaling pathways, which are closely associated with OXPHOS (Figure 6G). Furthermore, gene‐set enrichment (GSEA) analysis revealed that OXPHOS gene sets were enriched, while glycolysis was inhibited in the liver tissue of CD38 vaccine mice, which agreed with previous results (Figure 6H). Together, these results suggested that the CD38 peptide vaccine ameliorated abnormal metabolism‐related proteome shifts.

## 3 Discussion

Currently, vaccine‐based antiaging approaches are being increasingly developed (Yoshida et al. 2020; Pang et al. 2014; Suda et al. 2021). In this study, we demonstrated that an “ANYEFSQV”‐containing peptide vaccine could ameliorate aging‐associated phenotypes, specifically in metabolic dysfunction and physical and cognitive decline in mice. These beneficial effects are likely mediated by the elimination of CD38‐positive cells, although other mechanisms may be involved. CD38 vaccines also systematically shift the liver proteome during aging, specifically by increasing OXPHOS and inhibiting glycolysis‐associated biological processes.

CD38 is a type II transmembrane protein highly expressed in bone marrow and lymphoid tissues. The biological function of CD38 was first identified as a specific T cell‐activated marker, T10 (Terhorst et al. 1981). Subsequently, it was found to be expressed in monocyte‐derived cells (generally CD11b^+^), including inflammatory macrophages (Amici et al. 2018), dendritic cells (Fedele et al. 2004), and neutrophils (Fujita et al. 2005). Additionally, multifunctional enzyme activities, with NADase catalyzing β‐NAD and cyclase synthesizing cyclic adenosine diphosphoribose (cADPR), were identified (Liu et al. 2017, 2009; Malavasi et al. 2008; Shrimp et al. 2014). NAD^+^ is critical in the cellular and tissue aging process (Covarrubias et al. 2021). Indeed, we previously reported that increasing NAD^+^ levels via nicotinamide mononucleotide (NMN) administration restores the liver redox homeostasis and amends the protein acetylome (Luo, Ding, Zhu, et al. 2022; Luo, Ding, Yang, et al. 2022). Interestingly, the protective function of the CD38 vaccine partly overlapped with the functional pathways involved in these protective effects of NMN. Antiaging approaches aimed at NAD^+^ pool maintenance, including NAD^+^ precursor boosting, NAD^+^ synthesizing activation, and NAD^+^ consumption deceleration, also increase mice healthspan or reduce the risks for metabolic disorders, heart failure, and neurodegenerative diseases in vivo or in clinical trials (Roboon et al. 2021; Tarrago et al. 2018; Yoshino et al. 2021; Zhou et al. 2020; Yao et al. 2022; Peclat et al. 2022). Since CD38 is the key NAD^+^ consumption enzyme elevated with age, eliminating redundant CD38 is postulated to help balance the NAD^+^/NADH ratio, leading to protective physiological effects. CD38 also enzymatically produces cADPR and nicotinic acid adenine dinucleotide phosphate (NAADP), which act as double second messengers in Ca^2+^ signaling (Lee et al. 2022). In pancreatic beta cells, glucose induces increased intracellular Ca^2+^ via cADPR to secrete insulin (Okamoto 1999). Moreover, high serum insulin levels impair insulin sensitivity. Collectively, these results may partially account for the glucose and insulin tolerance in mice administered the CD38 vaccine.

We obtained RNA‐seq data deciphering cellular senescence and organ aging from the Aging Atlas. With aging, CD38 is highly expressed in the spleen, mesenteric fat, liver, etc. (Schaum et al. 2018; Wang et al. 2021; Schaum et al. 2020). Based on scRNA‐seq data, it is primarily enriched in immune cells, including lymphocytes and macrophages; however, its distribution varies among organs with aging (Sun et al. 2023). Previous studies have defined CD38‐expressing macrophages as driving age‐related NAD^+^ decline (Chini et al. 2020; Covarrubias et al. 2020). Using lentiviral systems, we also constructed CD38‐highly expressing RAW264.7 cells and found a markedly increased proportion of senescent cells in CD38^hi^ RAW264.7 (Figure S9A,B). Inhibition of CD38 activity restored the NAD^+^/NADH ratios and decreased ROS levels in CD38^hi^ RAW264.7 cells (Figure S9C,D). The expression of *Cd38* mRNA is also increased in the replicative‐senescent human diploid fibroblasts WI38 (Casella et al. 2019). Interestingly, our surface omics data also revealed that CD38 was enriched in aged mouse tail tip fibroblasts (Figure S10), partially explaining how CD38 antibodies prevented fibrosis in multiple organs (Shi et al. 2023). Interestingly, senescent/SASP markers (Suryadevara et al. 2024), including MMP9, ISG15, TNFRSF1B, CD36, ICAM1, NOTCH1, NOTCH3, and VIM, were decreased while the macrophage‐specific markers, including CD68, Fcgr1, and Fcgr4, were also decreased, in line with previous results (Figure S6). Previous studies have shown CD38 dictates age‐related NAD^+^ decline and mitochondrial dysfunction through SIRT3 (Camacho‐Pereira et al. 2016). In our proteomics data, mitochondrial proteins, including SIRT3, Parkin, TFAM, OPA1, and SOD2, were increased in the liver of CD38‐vaccine mice (Figure S6).

In this study, we report that the CD11b^hi^CD11c^mid^ cell proportions decreased upon CD38 vaccine administration. These cell subsets comprise most macrophages and monocytes, not T or B cells. Thus, our work reinforced previous reports that CD38^+^CD11b^hi^ myeloid cells accumulate with aging (Chini et al. 2020; Covarrubias et al. 2020). Using multicolor flow cytometry, we also found that the proportion of CD38^+^ CD8^+^ T cells in C57BL/6J mice (*n* = 6/group) at 20 months of age with prime‐boost CD38‐vaccine administration was slightly increased compared with KLH mice. This may be due to enhanced T cell‐mediated cellular immunity (Figure S2I). Proportions of splenic CD38^+^ CD4^+^ T cells, CD38^+^ CD19^+^ B cells, and liver CD38^+^ CD3^+^ T cells remain unchanged (Figure S2J–L). Considering CD38 is mainly expressed in LSCs and AML blasts, but not in HSCs, making it a relatively safe target (Marofi et al. 2021). Mice immunized with CD38 peptide vaccine showed less age‐related weight gain (Figure S7A,B). No differences in body temperature were observed between KLH mice and CD38‐vaccine mice (Figure S7C,D). To further evaluate the safety of vaccination, the complete blood counts (CBC) at 20 months were measured. No significant differences in white blood cells (WBC)/lymphoid cells/neutrophils/eosinophils/red blood cells (RBC) counts, mean corpuscular volume (MCV), and hemoglobin concentration were observed in CD38‐vaccine mice (Figure S7E–L). Notably, CD38‐vaccine decreases aspartate amino transferase (AST) and alanine aminotransferase (ALT), markers of liver damage in mice (Figure S7M,N). Future studies should focus on the effects of vaccination on longevity and pathogen‐host interaction.

Structurally, transmembrane CD38 can also be divided into type II orientation, with the short amino tail facing the extracellular environment, and type III orientation, with the catalytic domain facing the cytoplasm (Liu et al. 2017; Zhao et al. 2012). The topological reversal structure suggests that each motif of the CD38 protein has the potential to contain epitopes that can be processed either upon cell‐intrinsic antigen presentation or cross‐presentation. Peptide vaccines have the advantages of strong specificity and high safety and are highly suitable for targeted vaccine design. We identified “ANYEFSQV” as a T cell epitope for CD38 that elicits T cell‐specific responses. T cells play an indispensable role in adaptive immunity as functional performers of cellular immunity and auxiliaries of humoral immunity. A recent study revealed that cellular immunity after vaccination in the elderly is less effective in protecting against mutant strains of the new coronavirus, with weakened antigen presentation a major cause of the poor immune response (Xiao et al. 2023). In addition, the CD38 peptide vaccine may provide additional benefits for patients with congenital or acquired B‐cell deficiencies.

However, there are several limitations to the applications of CD38 vaccines. First, CD38 vaccines inevitably activate inflammatory responses, which may accelerate the aging process in the short term, given that the inflammatory response is thought to be a driving factor for cellular senescence. IL‐6 is a key senescence‐associated secretory cytokine that can be used as a quantitative indicator of the aging process. Hence, we first assessed serum IL‐6 levels in mice of different ages 3 weeks after CD38 vaccine or KLH administration. Young mice (8‐week‐old), middle‐aged mice (12‐month‐old), and aged mice (24‐month‐old) were immunized (Figure S8A). Serum IL‐6 levels were increased in middle and aged CD38 vaccine mice (Figure S8B). We then assessed serum IL‐6 levels in mice at a later time point following prime‐boost vaccination. Interestingly, serum IL‐6 levels were decreased in middle‐to‐aged CD38 vaccine mice, yet remained unchanged in young‐to‐mid CD38 vaccine mice (Figure S8C). The overall interpretation of these results was that a sufficient amount of CD38 in middle and aged mice elicited an immunogenetic peptide that initiated the cytotoxic process, releasing IL‐6. However, CD38 naturally mediates the production of several proinflammatory cytokines, including IL‐1β, IL‐6, and TNF‐α (Li, Liang, et al. 2022). Subsequently, the CD38 highly expressing cells accumulated in the tissues might have been continuously removed in the following months. Hence, further investigations are required to elucidate the long‐term safety of the CD38 vaccine and to elucidate how CD38 vaccines dynamically impact the immune system. Second, CD38 plays a role in myeloid cells in regulating responses to infection (Li, Li, et al. 2022; Pisu et al. 2024). Thus, it is also important to monitor the risk of infection in the future study. Third, “ANYEFSQV”‐containing peptides could be predicted from NetMHCpanII‐4.1 tools in our screening based on the immunogenicity, but did not appear in B cell epitope prediction tools. Therefore, other CD38 epitopes should be studied for multi‐epitope peptide vaccine design.

In conclusion, this study reports a proof‐of‐concept of a CD38 vaccine as a novel antiaging approach in mice. Further preclinical investigations using large animals and epitopes covering human CD38 are needed prior to clinical application. Future studies will also focus on its potential applications in other aging‐associated diseases, such as Alzheimer's disease.

## 4 Materials and Methods

### 4.1 Animals

All animal experimental procedures were reviewed and approved by the Laboratory Animal Research Center of Tsinghua University (Approval numbers: 22‐DHT3 and 23‐DHT6) and were performed in accordance with the guidelines and approval of the Institutional Animal Care and Use Committee (IACUC) of Tsinghua University. C57BL/6J wild‐type mice were purchased from the Jackson Laboratory at the Laboratory Animal Research Center of Tsinghua University. The mice were housed with *ad libitum* access to food and water in an environment maintained at 20°C–22°C with 30%–70% relative humidity and a 12‐h light/dark cycle. Male and female mice were used for physical performance assessment and metabolic monitoring at 15–17 months and 20 months. Male mice were used for cognitive assessment at 16 and 20 months of age.

### 4.2 Vaccine Development and Dosing Protocol

T cell epitopes were predicted using NetMHCpan4.1 and NetMHCIIpan‐4.0 algorithms (Reynisson et al. 2020) (https://services.healthtech.dtu.dk/service.php?NetMHCpan‐4.1), which screened MHC class I (H‐2Kb and H‐2Db) binding for all possible 8‐ to 11‐amino acid‐long sequences corresponding to mouse CD38. The predicted MHC class I binders were selected based on their relative ranking in NetMHCpan4.1. The peptides were synthesized by Anhui JYHX Co. Ltd. (Hefei, China). Each peptide was conjugated to keyhole limpet hemocyanin (KLH) and purified by reverse‐phase high‐performance liquid chromatography (HPLC) (> 95% purity). The KLH‐conjugated antigenic peptide (50 μg) and an equal volume of Alum Imject adjuvant (Thermo Fisher Scientific, USA) were emulsified according to the manufacturer's instructions. WT C57BL/6 mice at 2, 12, and 18 months old (*n* = 5–6/group) were subcutaneously injected with CD38 vaccines. In the control group, mice were injected with an equal volume of KLH mixed with the Alum Imject adjuvant.

### 4.3 Physical Activity Analysis

Murine physical activity was characterized by evaluating exercise endurance and motor function. To evaluate the exercise endurance, the total running distance was measured using a treadmill (Ugo Basile, Italy). Each mouse was acclimated to the treadmill for 20 min on three consecutive days at a speed of 10 m/min and a grade of 5%. After 1 day of rest, animals ran on the treadmill at an initial speed of 5 m/min and 5% gradient for 2 min, after which the speed was increased by 2 m/min every subsequent 2 min until the mice were exhausted. Exhaustion was the inability to remain on the treadmill despite an electrical shock stimulus.

To evaluate motor function, the total revolutions and maximal speeds normalized to the baseline speed when the mouse dropped off were measured using a rotarod (Ugo Basile, Italy). In the rotarod tests, each mouse was trained on the instrument at speeds of 5, 8, and 10 rpm for more than 200 s on Days 1, 2, and 3. During the test, the speed of the rotarod was increased from 5 to 40 rpm over a 5‐min interval.

### 4.4 Grip‐Strength Tests

To evaluate the strength of the mice, all‐paw grip strength (g) normalized to strength load (g)/body weight (g) was determined using a YLS‐13A Grip Strength Meter (Jinan Yiyan, China), with results averaged over 10 trials. For the hanging test, mice grabbed a 2‐mm‐thick metal wire 35 cm above the padded surface with their forelimbs only. Each mouse was acclimated to the equipment for 3 days of continuous training. The latency to fail was recorded and normalized to body weight as latency (s) × body weight (g), with the results averaged from two or three trials for each mouse.

### 4.5 Cognitive Function and Behavior Analysis

The Morris water maze test was used to assess the spatial learning and memory abilities of mice in each group. The water pool was divided into four quadrants (NW, NE, SW, and SE), and the platform was placed 1 cm below the water surface (Figure S3H). Before the experiments, mice who cannot swim continuously for more than 3 min were excluded. The experiment lasted 8 days. In hidden platform periods (the first 7 days), mice were placed in the water from the border of the four quadrants and trained to find the platform within 60 s or 120 s. The latency to the platform was recorded on the final day of the hidden platform period. On the final day, the platform was removed, and free exploration routes were recorded in a video. The cumulative distance to the zone where the mouse crossed the original platform in 120 s without placing the platform was calculated.

Mouse behavior was measured using the Open Field Test (OPFT). Individual mice were allowed to move freely within the box (60 × 60 × 60 cm) for 5 min, and the movements of each mouse were recorded. The apparatus was cleaned and sterilized between mice, and the experimental chamber was wiped with 75% alcohol. For novel object recognition tests, two objects (A and B) were placed within the box (area 1 and area 2, Figure S3I). Mice were put into the box for 10 min during 2 days of continuous training. The next day, object B was replaced by object C in area 2. Then mice were put into the box and the trajectory within the 5‐min exploration was recorded. The recognition index was calculated as the time of exploring new objects in area 2/(time of exploring new objects in area2 + time of exploring old objects in area 1). The distance walked and the average speed were analyzed using EthoVision XT 11.5 (Noldus Information Technology, Netherlands).

### 4.6 Frailty Assessment

A modified version of Whitehead method was used, which is noninvasive and reflexing aging‐related physical characteristics and the onset of aging traits in individual mouse (Sukoff Rizzo et al. 2018; Whitehead et al. 2013). Frailty index score is calculated as cumulative scores of 27 different measures as each given 0, 0.5, or 1 based on level of severity. Core body temperature and body weights were also recorded.

### 4.7 Metabolic Measurements

Mice were fasted for 16 h before intraperitoneal (i.p.) glucose tolerance tests (ipGTTs) were performed. Blood was collected from the tail vein before and 15, 30, 60, and 120 min after the i.p. injection of 2 g/kg glucose (Beyotime, China). Mice were fasted for 4 h before i.p. insulin tolerance tests (ITT) were performed. Blood was collected from the tail vein before and 15, 30, 60, and 120 min after the i.p. injection of 0.75 units/kg glucose (Beyotime). Glucose levels were measured using an ACCU‐CHEK Performa glucometer (Roche, Switzerland). Oxygen consumption, CO_2_ production, respiratory exchange ratio, energy expenditure, food consumption, water intakes, and locomotor activity were measured using a real‐time monitoring system for the metabolic cages (TSE, Germany).

### 4.8 Detection of Tissue NAD ^+^

Tissue NAD^+^ was extracted with pre‐chilled 80% methanol by a low‐temperature homogenizer and then plated in a refrigerator for 2 h at −80°C. Then, samples were centrifuged at 12,000 rpm for 20 min at 4°C, and each supernatant was dried by Speed‐vac at 4°C and dissolved in 80% methanol and analyzed by LC–MS/MS. The TSQ Quantiva Triple Quadrupole Mass Spectrometer with Ultimate 3000 (Thermo Fisher Scientific, Waltham, MA, United States) was employed for targeted quantitative analysis of NAD^+^. Retention time on the chromatograms and m/z were determined by Trace Finder software (version 3.2, Thermo‐Fisher Scientific). The NAD^+^/NADH ratio was measured using an NAD^+^/NADH Assay Kit with WST‐8 (Beyotime) according to the manufacturer's instructions.

### 4.9 Enzyme‐Linked Immunosorbent Assay (ELISA) and KSCN‐ELISA

IL‐6 levels were determined in mouse sera using the Mouse IL‐6 Precoated ELISA Kit (Dakewe Biotech Co. Ltd., Shenzhen, China) according to the manufacturer's instructions. The serum anti‐CD38 epitope antibody titer was quantified by ELISA. The plates were coated with bovine serum albumin (BSA)‐conjugated antigenic peptides (20 μg/mL; AnHui JYHX Co. Ltd., Hefei, China) diluted in 50 mM carbonate buffer overnight at 4°C. After blocking with phosphate‐buffered saline (PBS) containing 1% BSA, the sera were serially diluted from 50 to 3,276,800‐fold in PBS with 0.2% BSA, added to the plates, and incubated for 2 h.

For the KSCN‐ELISA assay, the following additional procedures were conducted. Double batch wells of biological repeats were prepared; to batch‐one wells, 0.5 M KSCN (Sigma‐Aldrich, USA) was added and incubated for 30 min at 37°C; to batch‐two wells, double‐distilled water (ddH_2_O) was added as the control for 30 min at 37°C.

For ELISAs and KSCN‐ELISAs, after washing each well with 0.02% PBS Tween‐20 (PBS‐T), the wells were incubated with horseradish peroxidase (HRP)‐conjugated antibodies specific for mouse IgG (1:1000; CST, USA) for 2 h at room temperature. After washing the wells with PBS‐T, color was developed with the peroxidase chromogenic substrate 3,3′,5,5′‐teteamethylbenzidine (TMB; Solarbio, China), and the reactions were terminated with 1 M hydrochloric acid. Absorbance was measured at 450 nm using a microplate absorbance reader, and the half‐maximal antibody titer was determined using GraphPad Prism 8. The standard curve to calculate the antibody response was prepared as previously described (Luo et al. 2025). The avidity index was defined as the serum dilution ratio giving half‐maximal binding (OD 50%) with or without the KSCN challenge.

### 4.10 ELISPOT Assay

ELISPOT assays for interferon (IFN)‐γ and IL‐4 were performed using the Mouse IFN‐γ Precoated and Mouse IL‐4 Precoated ELISPOT kits (Dakewe Biotech Co. Ltd.) according to the manufacturer's instructions. Briefly, splenocyte suspensions from immunized mice were added to the 96‐well ELISpot assay plates (10^6^ cells or 2 × 10^6^ cells per well) and stimulated with 10 μg/mL KLH protein or CD38 peptide‐1 synthesized by AnHui JYHX Co. Ltd. (Hefei, China), or PBS (Control) at 37°C overnight. The plates were washed with PBS and incubated with biotin‐conjugated antibodies for 2 h at room temperature. After washing, the plates were incubated with HRP‐conjugated streptavidin for 1 h at room temperature and subsequently with TMB substrate. Colored spots were photographed using a dissecting microscope and counted with an AID Elispot reader (AID, Germany).

### 4.11 RNA Extraction and Real‐Time Quantitative PCR

For quantitative PCR, total RNA was extracted from mouse liver samples with a low‐temperature homogenizer using TRIzol reagent (Tiangen, China). cDNA was synthesized with a commercial cDNA reverse transcription kit (Cwbio, China) following the manufacturer's instructions. Quantitative real‐time PCR was performed using SYBR Green Reaction Mixture (Cwbio) and analyzed with the Roche LightCycler 96 System (Roche, Switzerland). Mouse β‐actin (*Actb*) was used as an internal control for relative quantification. The primers used for the qPCR are listed in Table S1.

### 4.12 Histology and Immunohistochemistry

Tissues were fixed with 4% paraformaldehyde overnight. They were then embedded in paraffin, sliced into 5‐μm sections, deparaffinized, and hydrated sequentially. For histological analysis, the sections were stained with hematoxylin and eosin. For immunohistochemical analysis, antigen retrieval was performed using ethylenediaminetetraacetic acid (EDTA) buffer at pH = 9.0. Endogenous peroxidase was blocked with 3% BSA at room temperature for 30 min before incubation with primary antibodies (anti‐mouse CD38 rabbit pAb and anti‐mouse p21 rabbit mAb antibody; GB114832 and GB155313, Servicebio, China) at 4°C overnight. After washing twice with PBS, the slides were incubated with the secondary antibody (S‐vision poly HRP‐conjugated Goat Anti‐Rabbit IgG; Servicebio, China). The signal was detected by 3,3′‐diaminobenzidine (DAB) staining. The nuclei were stained with hematoxylin. Images were captured with Eclipse C1 Microscope System (Nikon, Japan).

### 4.13 Immunofluorescence

After deparaffinization and rehydration of sections, antigen retrieval was performed by boiling slides in 1× citrate buffer (pH 6.0). The slides were blocked in 3% BSA for 30 min. Anti‐F4/80 antibody (Rabbit pAb, 1:1000, GB113373, Servicebio), anti‐CD38 antibody (Rabbit pAb, 1:1000, GB114832, Servicebio), and anti‐CD11b antibody (Rabbit mAb, 1:1000, GB15058, Servicebio) were used as primary antibodies (4°C overnight incubation). Slides were washed 3 times with 1× PBS, followed by a 50 min incubation with HRP‐conjugated goat‐anti‐rabbit IgG (1:500, GB23303, Servicebio). After washing, iF488‐Tyramide (1:500, G1231, Servicebio) or CY3‐Tyramide (1:500, G1223, Servicebio) was used (10 min staining in dark), followed by TBST washing and citrate‐mediated antigen retrieval. Then, another round of antibody incubation and fluorescence staining was performed. Finally, nuclei were stained with DAPI (G1012, Servicebio). Images were captured with the Eclipse E100 Microscope System (Nikon, Japan).

### 4.14 SA‐β‐Galactosidase Staining

SA‐β‐Gal staining was carried out with an SA‐β‐Gal staining kit (Beyotime) according to the manufacturer's instructions. Briefly, to assess SA‐β‐Gal activity across tissues, 20‐μm frozen sections or cultured cells were fixed with β‐galactosidase staining fixation solution for 1 h, washed with PBS twice, and incubated with SA‐β‐Gal staining solution at 37°C overnight. The samples were then imaged using light microscopy (Nikon‐90i, Japan) or Axioscan Z1 microscope slide scanner (ZEISS, Germany), and blue‐stained cells were identified as an event of cellular senescence.

### 4.15 Cell Isolation and Flow Cytometry

Spleen samples from immunized mice were triturated and suspended in MACS buffer (PBS supplemented with 1% FBS and 5 mM EDTA; pH = 7.4). The suspension was passed through a 70‐μm filter, centrifuged at 1000 × g and 4°C for 10 min, and resuspended in MACS buffer. The single cell suspension of liver was prepared using a similar protocol described in previous studies (Li et al. 2025). Red blood cells were removed from cell suspensions using ACK erythrocyte‐lysing buffer (150 mM NH_4_Cl, 10 mM KHCO_3_, and 0.1 mM Na_2_EDTA; pH 7.4). After lysing the erythrocytes, the suspended splenocytes were passed again through the 70‐μm filter, centrifuged at 1000 × g and 4°C for 10 min, and resuspended in MACS buffer. After blocking Fc‐receptors with an anti‐mouse CD16/32 antibody (1 μg per million cells in 100 μL, 553141, BD, USA) for 20 min at 4°C, cells were stained with a mixture of fluorescently labeled antibodies at 4°C for 40 min in the dark. The antibodies used were specific for BV711‐CD19 (1:100, 563038, BD, USA), PerCPcy5.5‐CD3 (1:100, 560527, BD, USA), FITC‐CD4 (1:100, 100406, BioLegend, USA), PEcy7‐CD8 (1:100, 100722, BioLegend, USA), PacificBlue‐CD38 (1:100, 102720, BioLegend, USA), APC‐CD11b (1:100, 101212, BioLegend, USA), and APC‐cy7‐CD11c (1:100, 117324, BioLegend, USA). CellEvent Senescence Green Kit (C10840, Thermo, USA) was used to detect SPiDER‐βGal^+^ senescent cells after fixing the samples. Flow cytometry was performed using BD LSRFortessa (BD, USA), and the results were analyzed with FlowJo v10.

### 4.16 Blood Analysis

For whole mouse blood analysis, 50 μL blood was freshly collected from tail vein into a 1.5 mL tube precoated with EDTA‐K2 anticoagulant (BSH00569, LabTools). Complete blood counts and hemoglobin concentrations were analyzed by an automated compact 6‐part differential hematology analyzer (Sysmex XN‐L‐550, Japan). Serum ALT and AST levels were determined with Alanine aminotransferase Assay and Aspartate aminotransferase Assay (C009‐2‐1, C010‐2‐1, Nanjing Jiancheng, China) according to the manufacturer's instructions.

### 4.17 Construction of CD38 Overexpression RAW264.7 Cell Line and Cellular ROS Tests

CDS region of mouse CD38 was subcloned into lentivirus vector pLVX‐IRES‐ZsGreen1 to construct a lentiviral vector pLVX‐mouseCD38‐IRES‐ZsGreen1. Next, recombinant construct with three helper plasmids pLP1, pLP2, and VSVG were transiently transfected into 293T cells using PEI to collect lentivirus particles. Empty pLVX‐IRES‐ZsGreen1 was used as a negative control. Lentivirus particles were concentrated with 4 M NaCl and PEG6000 and then used to infect RAW264.7 cells with 10 ng/μl polybrene. GFP positive polyclone cells were sorted by flow cytometer to generate stable cell lines. Cells were cultured in DMEM (Wisent, Canada) with 10% fetal bovine serum (Wisent, Canada) and 1% penicillin/streptomycin (Wisent, Canada). 500 μM NMN (Huateng pharma, China) and 10 μM 78c (Macklin, China) were used to treat cells for 24 h. Cellular reactive oxygen species (ROS) levels were detected using the CellROX Deep Red Reagents (Thermo Fisher Scientific, Waltham, MA) according to the manufacturer's protocol.

### 4.18 Tandem Mass Tag (TMT) Quantitative Proteomic Analysis by LC–MS/MS

Proteomic analyses were performed as described previously (Wang et al. 2024). Briefly, liver tissues from individual mice were lysed by RIPA lysis buffer (Beyotime, China) supplemented with a protease inhibitor cocktail. Supernatant collected from sonicated protein lysate was added to acetone in a 1:5 vol at −30°C for precipitation and then dissolved with 8 M urea. Next, 150 μg of protein per sample was digested with Trypsin Gold, Mass Spectrometry Grade (Promega, USA) at 37°C overnight for 16 h. Tryptic peptides were desalted through a Sep‐Pak C18 Cartridge and labeled with equal TMT 6‐plex reagents (Thermo Fisher Scientific, USA). The mixed‐label peptides were separated by reverse‐phase chromatography with a secondary desalting procedure and subjected to LC–MS/MS analysis. Labeled peptides were injected onto a UHPLC 3000 system coupled to a Thermo Scientific Orbitrap Ascend Tribrid mass spectrometer using a C‐18 analytical column (300 Å, 5 μm, Thermo Fisher Scientific, USA). The peptides were eluted using a gradient elution program at a 0.300 μL/min flow rate. Mobile phase A comprised 0.1% formic acid, and mobile phase B comprised 100% acetonitrile and 0.1% formic acid. The mass spectrometer was operated in data‐dependent acquisition (DDA) mode using Xcalibur 4.5.445.18 software. MS1 spectra were acquired in the mass range of 300–1800 m/z at a 60,000 resolution. The spray voltage was set to 2100 V, and the Automatic Gain Control (AGC) target was set to 3e^6^. For MS2 scans, the top 40 most intense precursor ions were fragmented in the HCD collision cell at a normalized collision energy of 32% using a 0.4 Da isolation window. The dynamic exclusion duration was set to 15 s, the AGC target was set to 1e^5^, and the maximum injection time was set to 100 min. Raw mass spectrometry data were searched against the *Mus musculus* database using Proteome Discoverer 2.3 software.

### 4.19 Statistical Analyses

Values are presented as the mean ± standard error of the mean (SEM) or standard deviation (SD). The statistical significance of the differences between two groups was assessed using a two‐tailed unpaired *t*‐test. Differences among multiple groups were assessed using analysis of variance (ANOVA), Tukey's or Sidak's multiple comparison test. Analysis of covariance (ANCOVA) was used to correct the covariance effects of body weight in metabolic cage analysis. A regression analysis plot was made in CalR2 (https://calrapp.org/) (Mina et al. 2018). Differences were considered statistically significant at *p* < 0.05. Statistical analyses were performed using GraphPad Prism, version 8 (GraphPad Software). R Studio was used to create volcano plots and heat maps.

## Author Contributions

**Shangcheng Yu:** conceptualization, data curation, formal analysis, investigation, methodology, resources, validation, visualization, writing – original draft, writing – review, and editing. **Zhiqiang Li:** data curation and visualization of tissue β‐gal staining. **Yuxiang Tang:** data visualization in bioinformatics analysis and image layout. **Yuling Chen:** LC–MS/MS analysis. **Yingying Ma:** surface‐omics in TTFs. **Kunlin Du:** assisting with ELISA and GTT experiments. **Zhaoyun Zong** and **Kangze Feng:** investigation. **Yali Wei:** CBC analyses. **Limeng Chen:** discussion. **Haiteng Deng:** conceptualization, funding acquisition, methodology, project administration, supervision, validation, writing – review, and editing.

## Conflicts of Interest

The authors declare no conflicts of interest.

## Supporting information


Figures S1–S10.

Table S1.

Table S2.

Table S3.

## Data Availability

All the data supporting the findings of this study are available from the corresponding author upon request.
