# Supplementary material for: CD38‐Targeting Peptide Vaccine Ameliorates Aging‐Associated Phenotypes in Mice
Source: Aging Cell. 2025 Jun 25;24(9):e70147. doi: 10.1111/acel.70147 (PMC12419845; doi:10.1111/acel.70147)
Supplement: Supplementary file 1 — Figures S1–S10. [file ACEL-24-e70147-s004.docx]

Supplementary Materials:

**
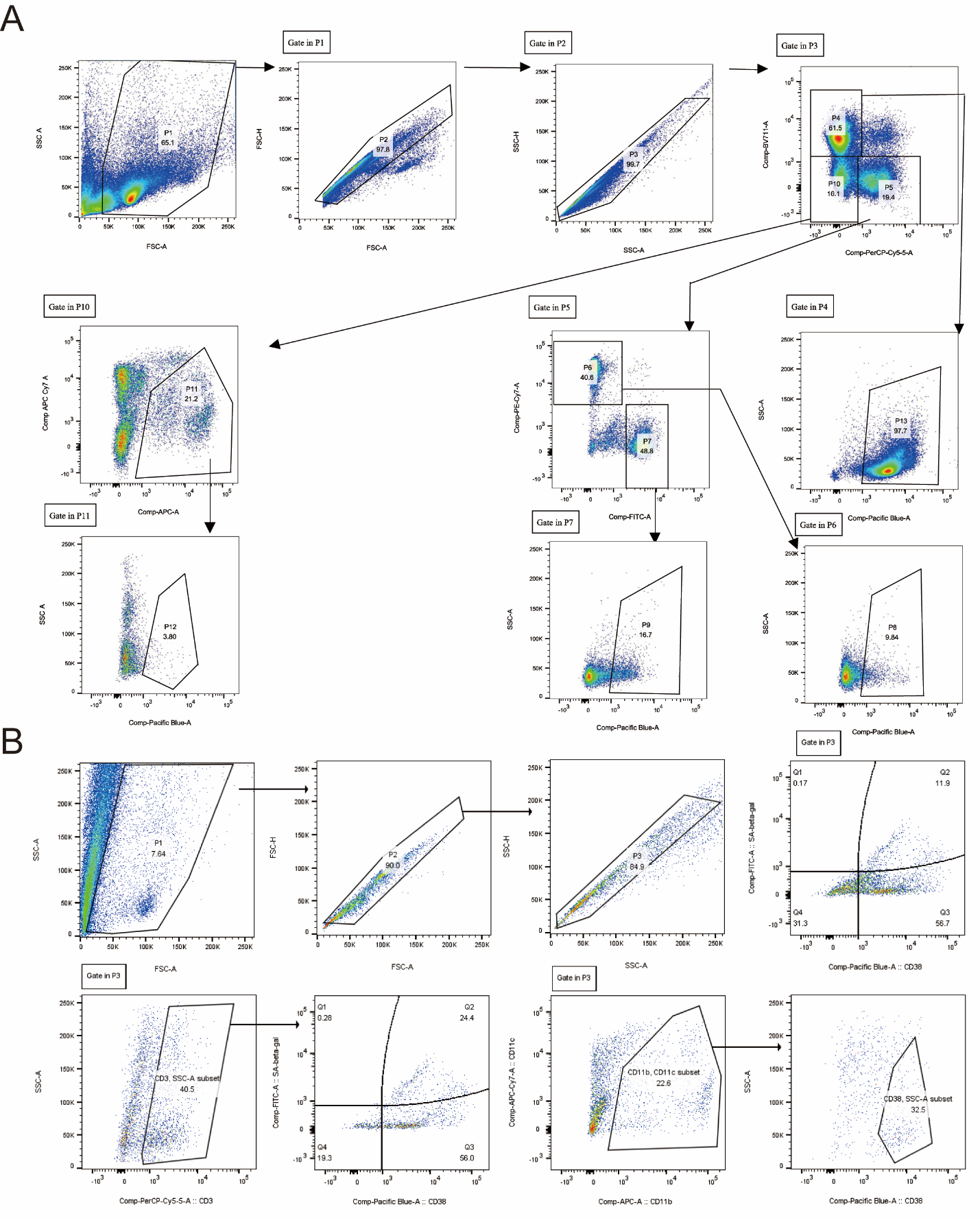
**

**Figure S1.** Multicolor flow cytometry gating strategy. (**A**)Multicolor flow cytometry gating strategy for splenocyte analysis. For phenotypic analysis of major splenic macrophages, T cells, and B cells, a multi-color staining panel (BV711-CD19, PerCPcy5.5-CD3, FITC-CD4, PEcy7-CD8, PacificBlue-CD38, APC-CD11b, APC-cy7-CD11c) was applied using the following gating strategy: (1) Gate P3: FSC and SSC excluded debris, cell doublets, and dead cells. (2) CD3 × CD19 discriminate between B cells (Gate P4) and T cells (Gate P5), i.e., major splenocyte subsets. (3) CD11b × CD11c: for gating CD19^-^ CD3^-^ CD11b^hi^ CD11c^mid^ cells. (4) CD38^+^ cells proportion in each gate. (**B**) Multicolor flow cytometry gating strategy for analyzing mouse liver with staining panel (PerCPcy5.5-CD3, FITC-SPiDER-β-gal, APC-CD11b, APC-cy7-CD11c, PacificBlue-CD38). FSC and SSC were used to gate single cells as shown. (1) CD38 × SPiDER-β-gal was used for gating total CD38^+^ cells (Q2+Q3) and senescent cells (Q1+Q2) (2) CD3 × SSC-A was used for gating T cells and CD38^+^ cells (Q2+Q3) inside the gate (3) CD11b × CD11c was used for gating CD11b^hi^ CD11c^mid^ cells and CD38^+^ cells inside last gate.


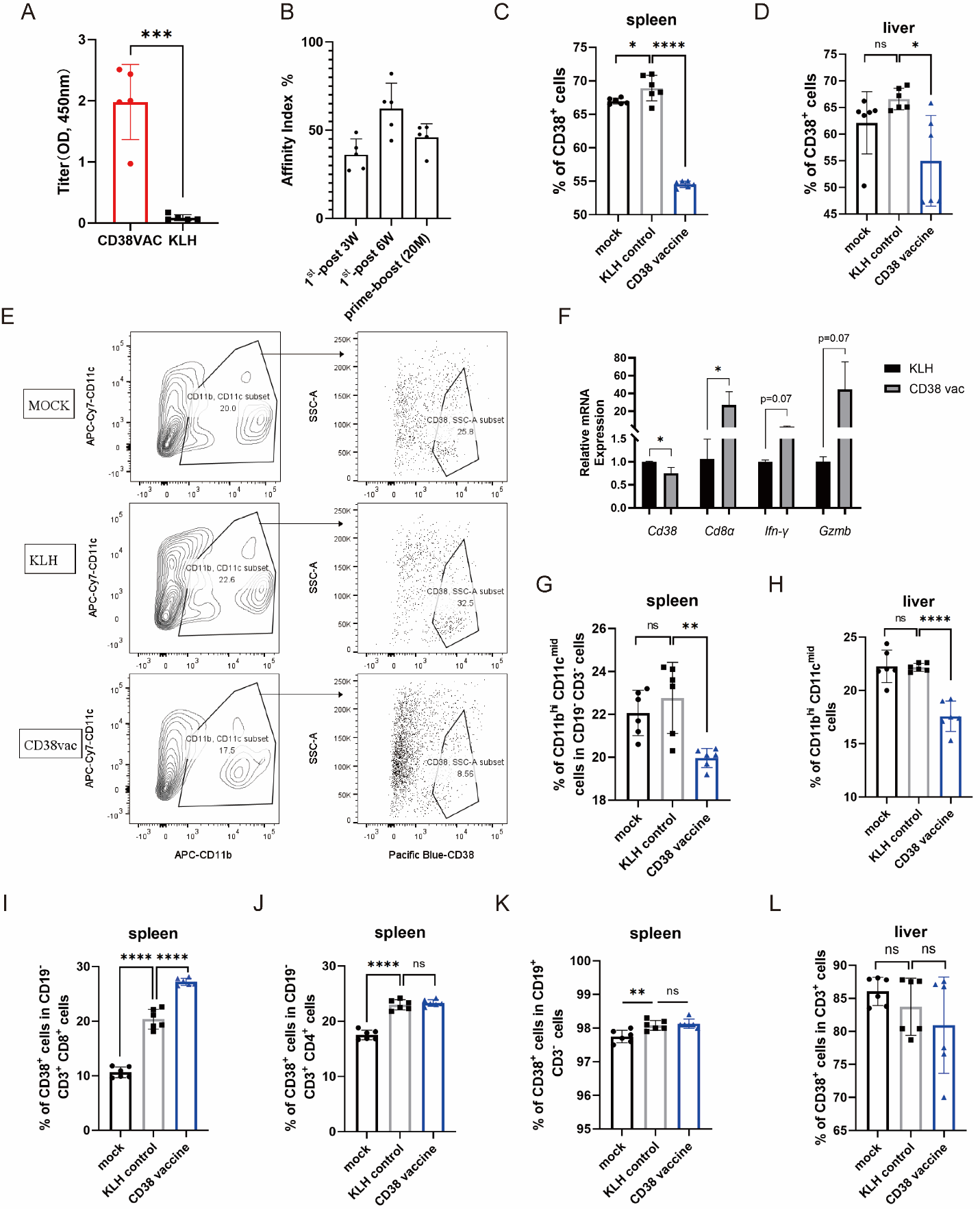


**Figure S2.** Characterization of immune response to CD38 vaccine. Related to Figure 2. **(A)** Analyses of mice serum at the end of the prime-boost vaccination protocol (20-month-old). Serum dilution at 4,050-fold were measured by ELISA (n = 5/group, each dot represents an individual mouse). (**B**) Avidity index of CD38-peptide specific antibody in mice at 3 or 6 weeks post first immunization and at the end of the prime-boost vaccination protocol (*n* = 5/group, paired for ratio calculation). (**C-D**) Proportion of CD38^+^ cells in the splenic (C) and liver(D) tissues of male C57BL/6J mice (*n* = 6/group) at 20 months old with or without prime-boost KLH or CD38-vaccine administration. (**E**) FCS illustration of liver CD38^+^ CD11b^hi^ CD11c^mid^ cell propotions in male C57BL/6J mice at 20 months old with or without prime-boost KLH or CD38-vaccine administration. (**F**) Splenic mRNA levels of Th1-related genes, determined by qRT-PCR (n = 3 mice/group) at the end of the prime-boost vaccination protocol. (**G-H**) Proportion of CD11b^hi^ CD11c^mid^ cells in CD19^-^CD3^-^ cells in the splenic tissues (G) and proportion of CD11b^hi^ CD11c^mid^ cells in the liver tissues (H) of male C57BL/6J mice (*n* = 6/group) at 20 months old with or without prime-boost KLH or CD38-vaccine administration. (**I-K**) Proportion of CD38^+^ cells in CD19^-^CD3^+^CD8^+^ cells (I)(P8 in Gate P6), CD19^-^CD3^+^CD4^+^ cells (J)(P9 in Gate P7). CD19^+^CD3^-^ cells (K)(P13 in Gate P4) in the splenic tissues of male C57BL/6J mice (*n* = 6/group) at 20 months old with or without prime-boost KLH or CD38-vaccine administration. (**L**) Proportion of CD38+ cells in CD3^+^ cells in the liver tissues of male C57BL/6J mice (*n* = 6/group) at 20 months with or without prime-boost KLH or CD38-vaccine administration. All data were analyzed by two-tailed Student’s *t*-test (A, F) and ANOVA followed by Sidak's multiple comparisons test (C, D, G, H, I, J, K, L); values represent the mean ± SD; **p* < 0.05, ***p* < 0.01, ****p* < 0.001, *****p* < 0.0001, ns = not significant.


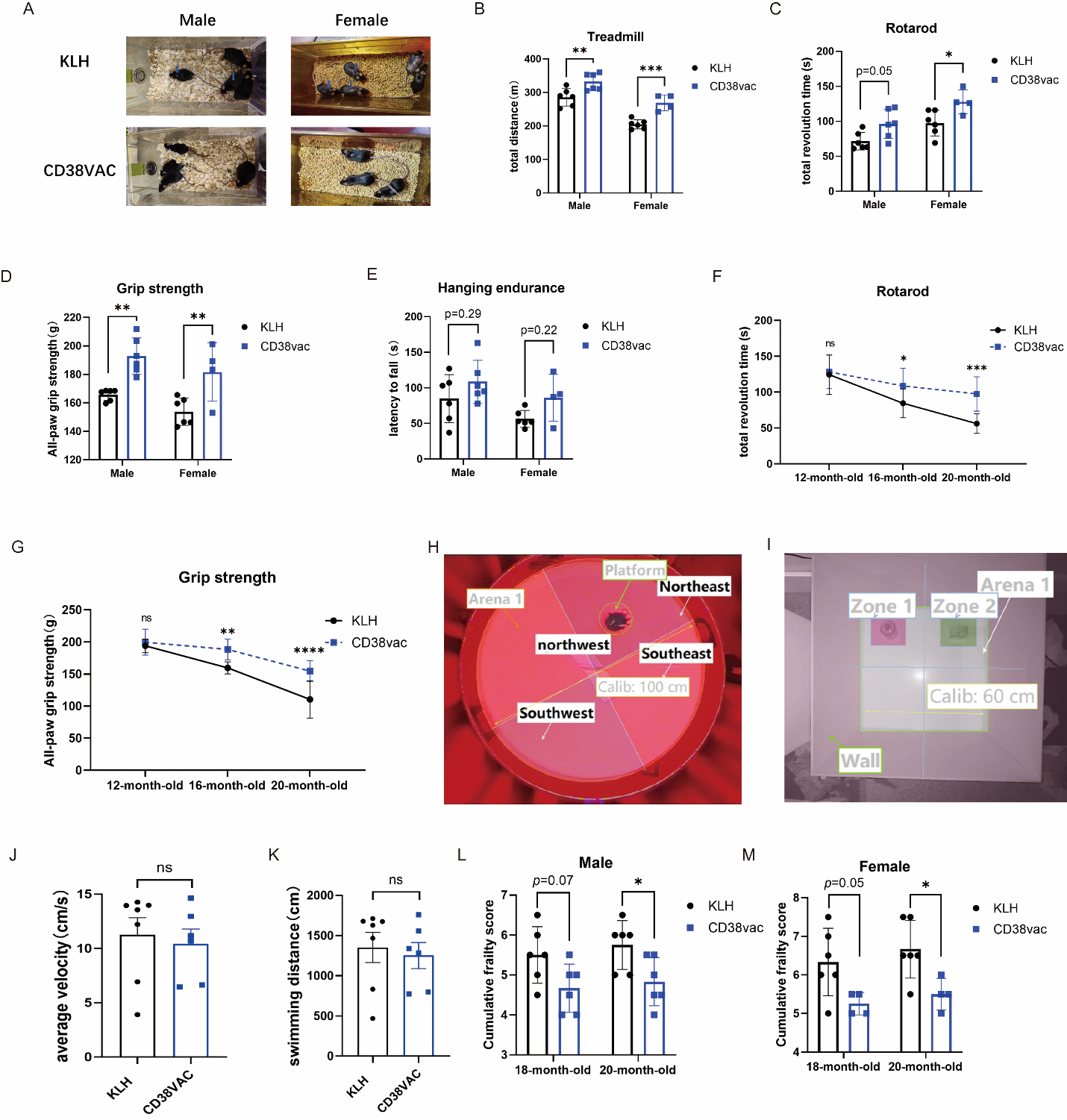


**Figure S3.** CD38 vaccine prevents physical and cognitive decline in naturally-aged mice. Related to Figure 3. (**A**) The appearance of KLH mice and CD38-vaccine mice at 20 months old.

(**B–E**) Total walking distance on treadmill (B), total revolution time on rotarod (C), all-paw grip strength (D) and hanging endurance (E) of KLH mice and CD38-vaccine mice at 15–16 months old (Male: *n* = 6, 6; Female: *n* = 6, 4). (**F–G**) Rotarod performance (F) and grip-strength (G) of KLH mice and CD38-vaccine mice at 12, 16, and 20 months old (male and female, *n* = 12, 10). (**H**) Swimming pool setup for the Morris water maze. The pool was divided into four quadrants. Platform was located in the northwest quadrant. (**I**) Object positions in the box: Old object: Zone1. Novel object: Zone 2. (**J-K**) Average velocity (J) and swimming distances (K) of male KLH mice and CD38-vaccine mice (n = 7, 6; at 16 months old) during 2-min MWM exploration (*n* = 7, 6). (**L-M**) Cumulative frailty score of male (L) and female (M) KLH mice and CD38-vaccine mice at 18 and 20 months old (Male: *n* = 6, 6; Female: *n* = 6, 4). All data were analyzed by two-tailed Student’s t-test; values represent the mean ± SD; *p < 0.05, **p < 0.01, ***p < 0.001, ****p < 0.0001, ns = not significant.


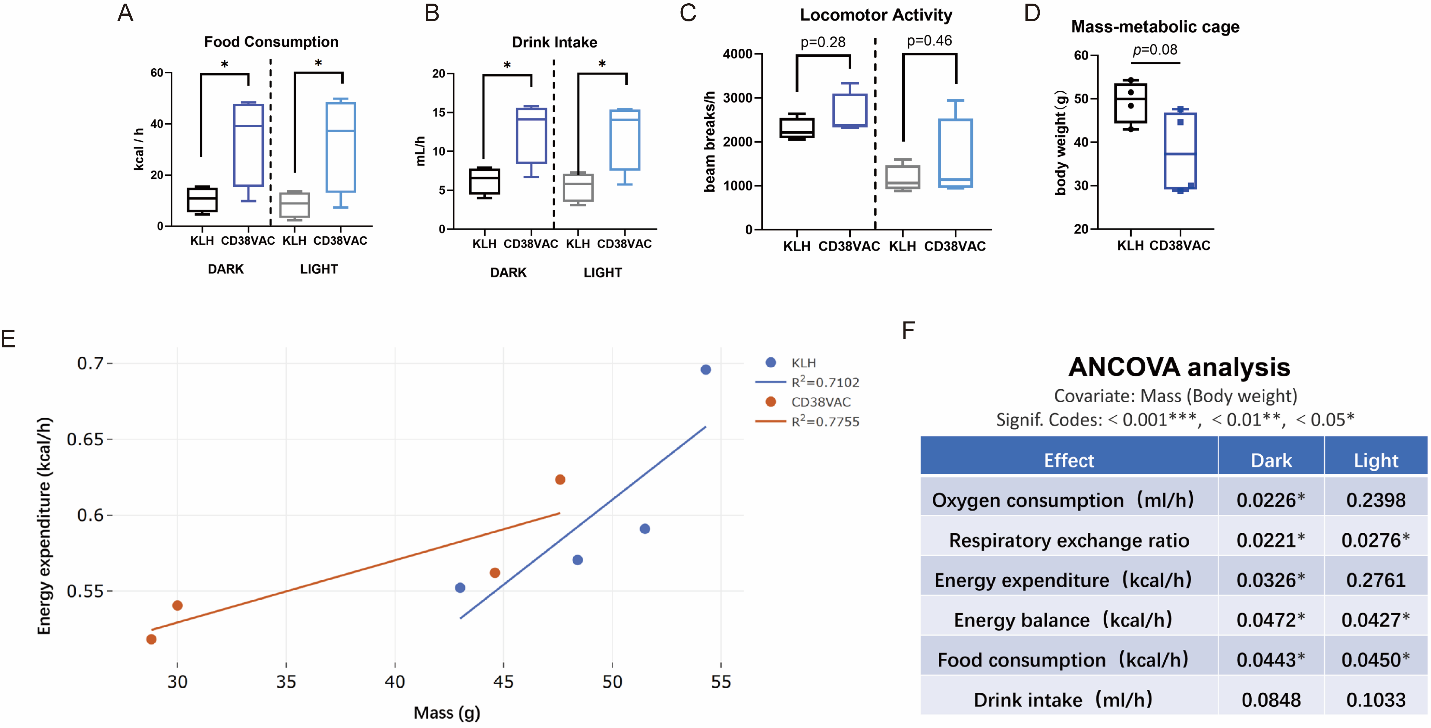


**Figure S4.** CD38 peptide vaccine ameliorates metabolic dysfunction-associated features in naturally-aged mice. Related to Figure 4. (**A-D**) Food consumption (A), drink intake (B), and locomotor activity (C) and weight (D) of KLH mice and CD38-vaccine mice at 17 months old. Data are shown as box-and-whisker plots, where a box extends from the 25th to 75th percentile with the median shown as a line in the middle, and whiskers indicate the smallest and largest values; Data were analyzed by two-tailed Student’s t-test; *p < 0.05. (**E**) Plot of ANCOVA predicted mean energy expenditure for total body weight values in KLH mice and CD38-vaccine mice at 17 months old (*n* =4, 4). Multiple linear regression analysis of the impact of body mass covariate on energy expenditure was calculated using the CalR2. (**F**) The group adjusted *p*-values of oxygen consumption, RER, EE, energy balance, food consumption and drink intake during dark cycle and light cycle. *p*-values referred to significance of the ANCOVA-adjusted comparison when body weight were taken over between two groups.


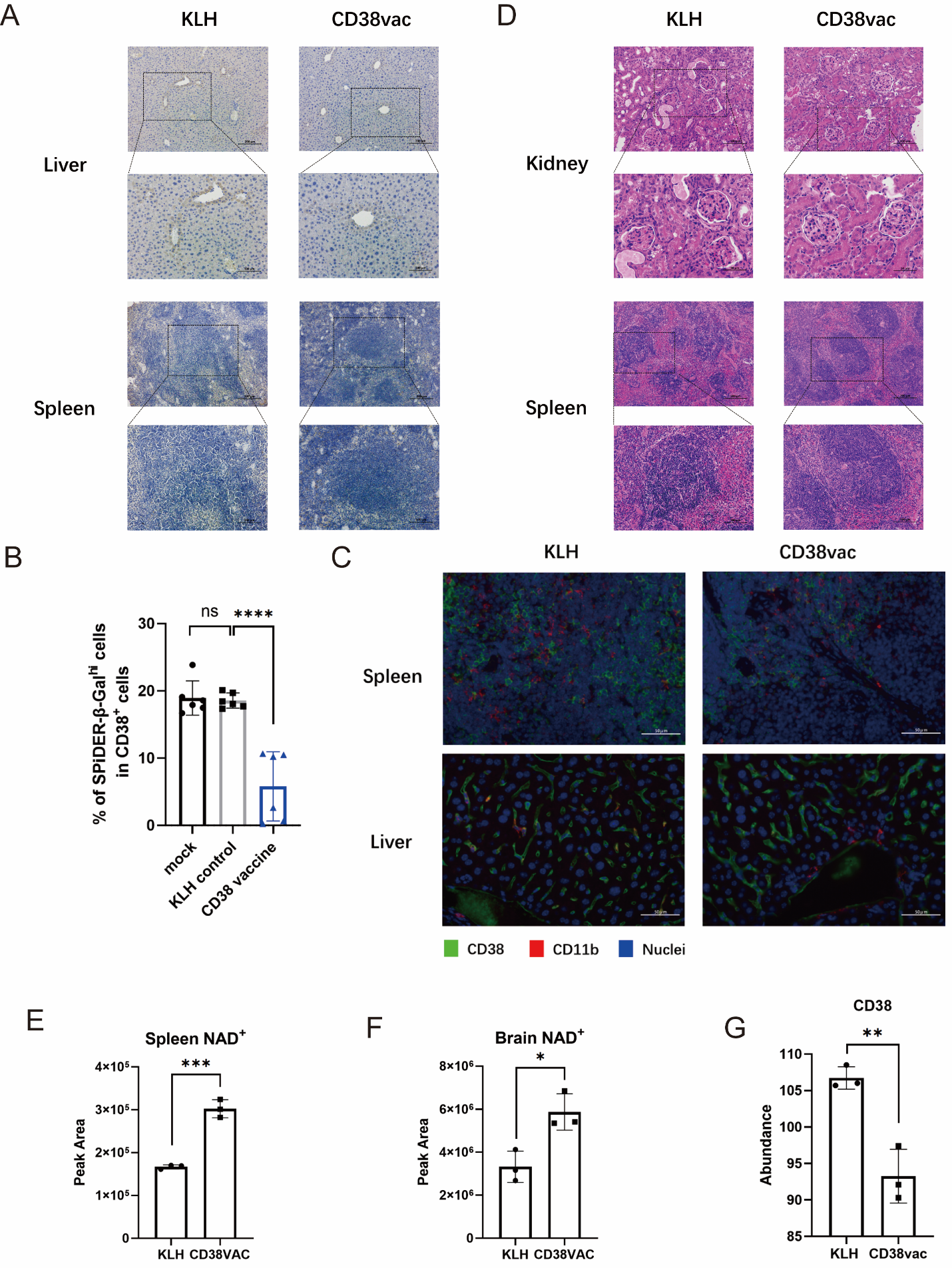


**Figure S5.** Decreased senescent cells and elevated NAD^+^ in tissues of naturally-aged mice following CD38-peptide vaccination. Related to Figure 5. (**A**) Immunohistochemical staining of mouse p21 in liver and spleen tissue obtained from KLH mice and CD38 vaccine mice. Scale bars, 200 μm (up) and 100 μm (down). (**B**) The proportions of SPiDER-β-gal^+^ cells in CD38^+^ cells [Q2/(Q2+Q3)] in liver tissues of male C57BL/6J mice (n = 6/group) at 20 months old with or without KLH or CD38 vaccine. (**C**) Immunofluorescence staining of CD38 (green), myeloid cells marker antigen CD11b (red) and nuclei with DAPI (blue) in spleen and liver from KLH mice or CD38 vaccine mice (20-month-old), representative of n = 3/group. Scale bar: 50 μm. (**D**) Representative images of hematoxylin and eosin (H&E)-stained kidney and spleen sections. Kidney image: scale bars, 100 μm (up) and 50 μm (down); Spleen image: scale bars, 200 μm (up) and 100 μm (down) (*n* = 6/group). (**E-F**) LC–MS quantification of NAD^+^ in the spleen and brain from KLH mice and CD38-vaccine mice at 20 months old (n = 3 mice/group). (**G**) Proteomic identification of CD38 protein expression level in the liver tissue of KLH mice and CD38 vaccine mice at 20 months old. Related to Figure 6. All data were analyzed by ANOVA followed by Sidak's multiple comparisons test (B) and two-tailed Student’s *t*-test (E, F, G); values represent the mean ± SD; **p* < 0.05, ***p* < 0.01, ****p* < 0.001, *****p* < 0.0001, ns = not significant.


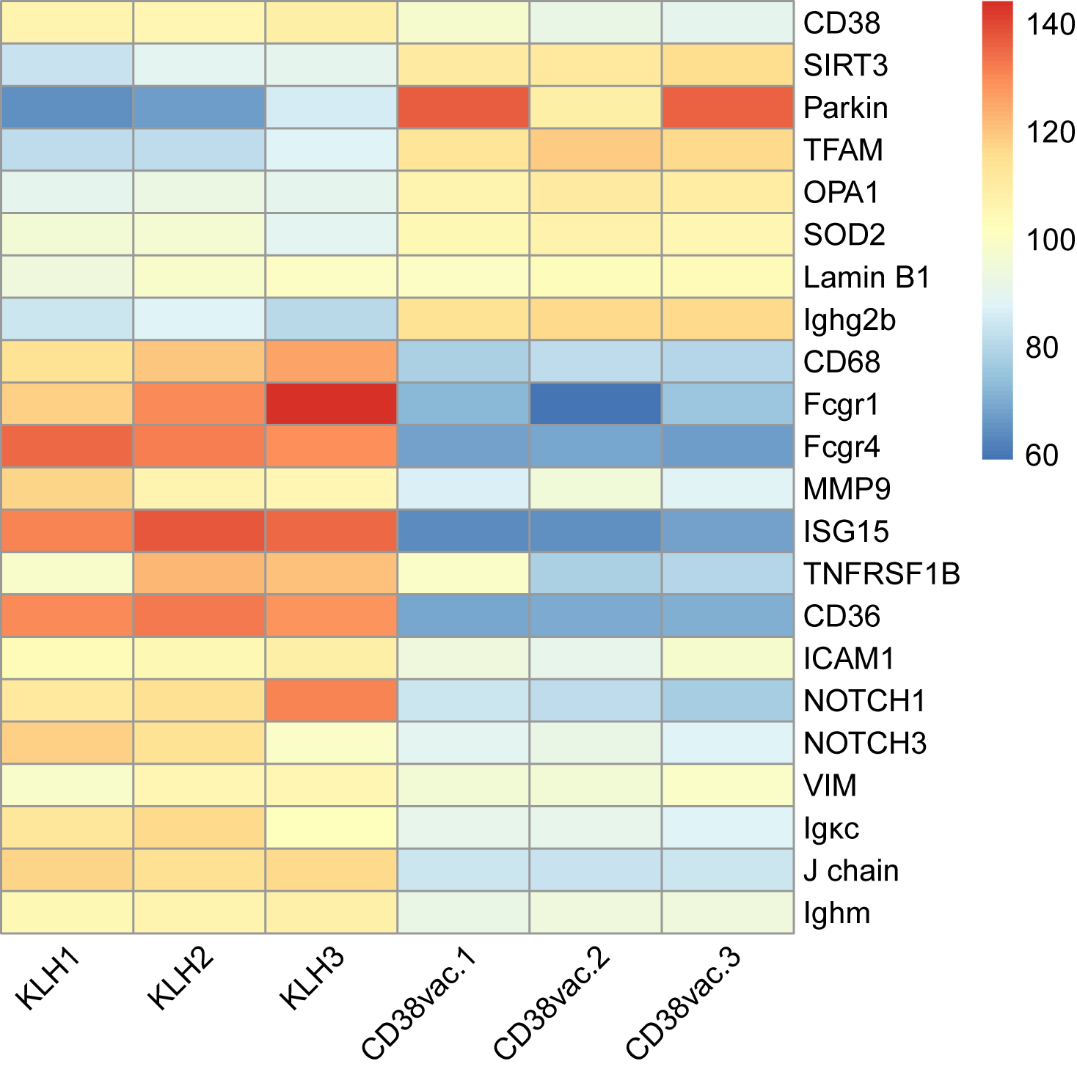


**Figure S6.** CD38 peptide vaccine restores liver proteome. Related to Figure 6. Heatmaps for selected protein expression levels in liver of KLH mice and CD38-vaccine mice.


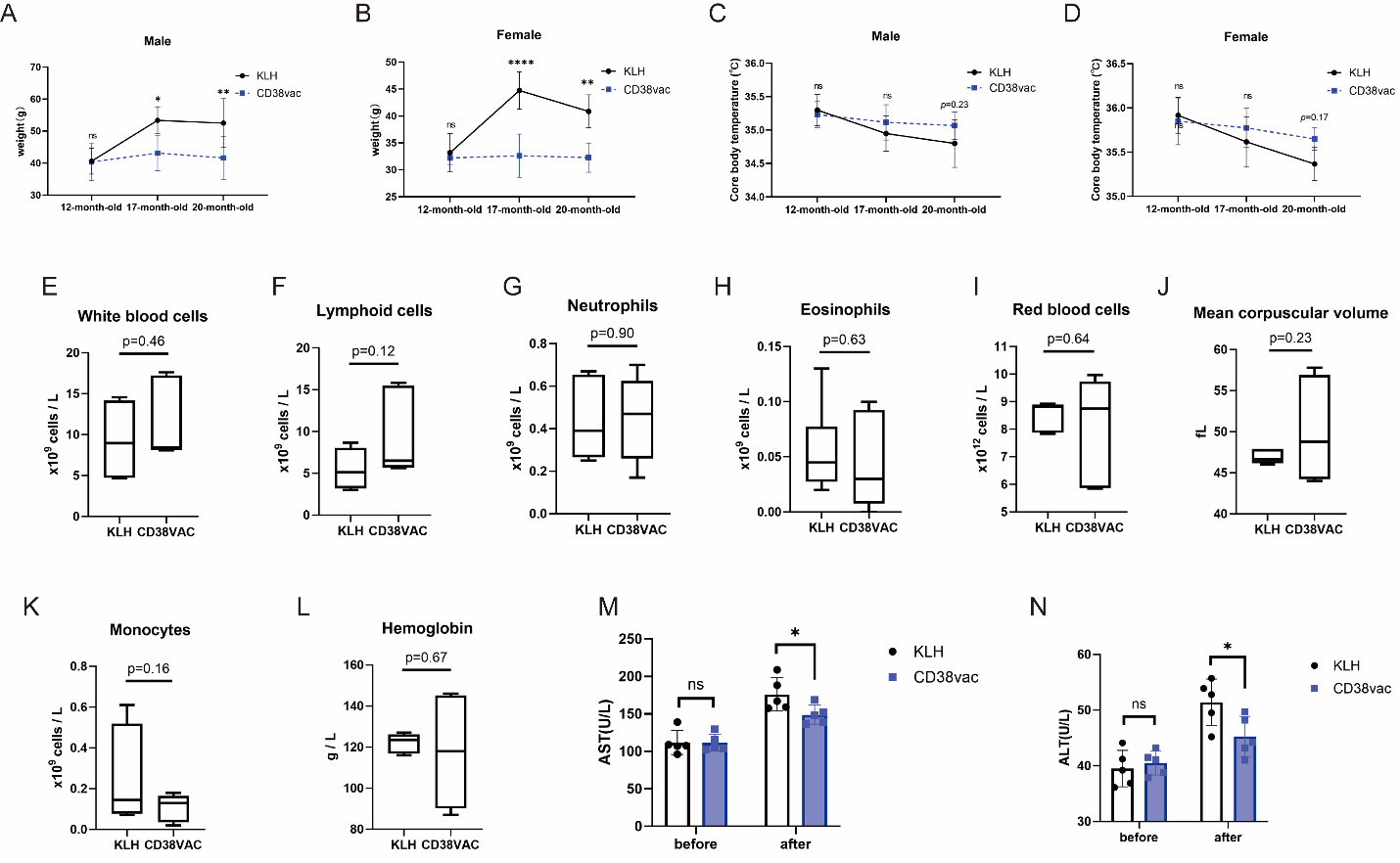


**Figure S7.** Safety assessment of CD38 peptide vaccine. (**A-D**) Body weight and core body temperature of KLH mice and CD38-vaccine mice at 12, 17, and 20 months old (Male: *n* = 6, 6; Female: *n* = 6, 4) (**E-K**) Complete blood counts (CBC) [including white blood cells (E), lymphoid cells (F), neutrophils (G), eosinophils (H), red blood cells (I), mean corpuscular volume (J), monocytes (K)] of male KLH mice and CD38-vaccine mice at 20 months old (*n* = 6, 6). (**L**) Blood hemoglobin concentrations of male KLH mice and CD38-vaccine mice at 20 months old (*n* = 6, 6). (**M-N**) Serum AST and ALT levels in male KLH mice and CD38-vaccine mice at 20 months old (*n* = 5, 5). All data were analyzed by two-tailed Student’s t-test; values represent the mean ± SD; *p < 0.05, **p < 0.01, ****p < 0.0001, ns = not significant.


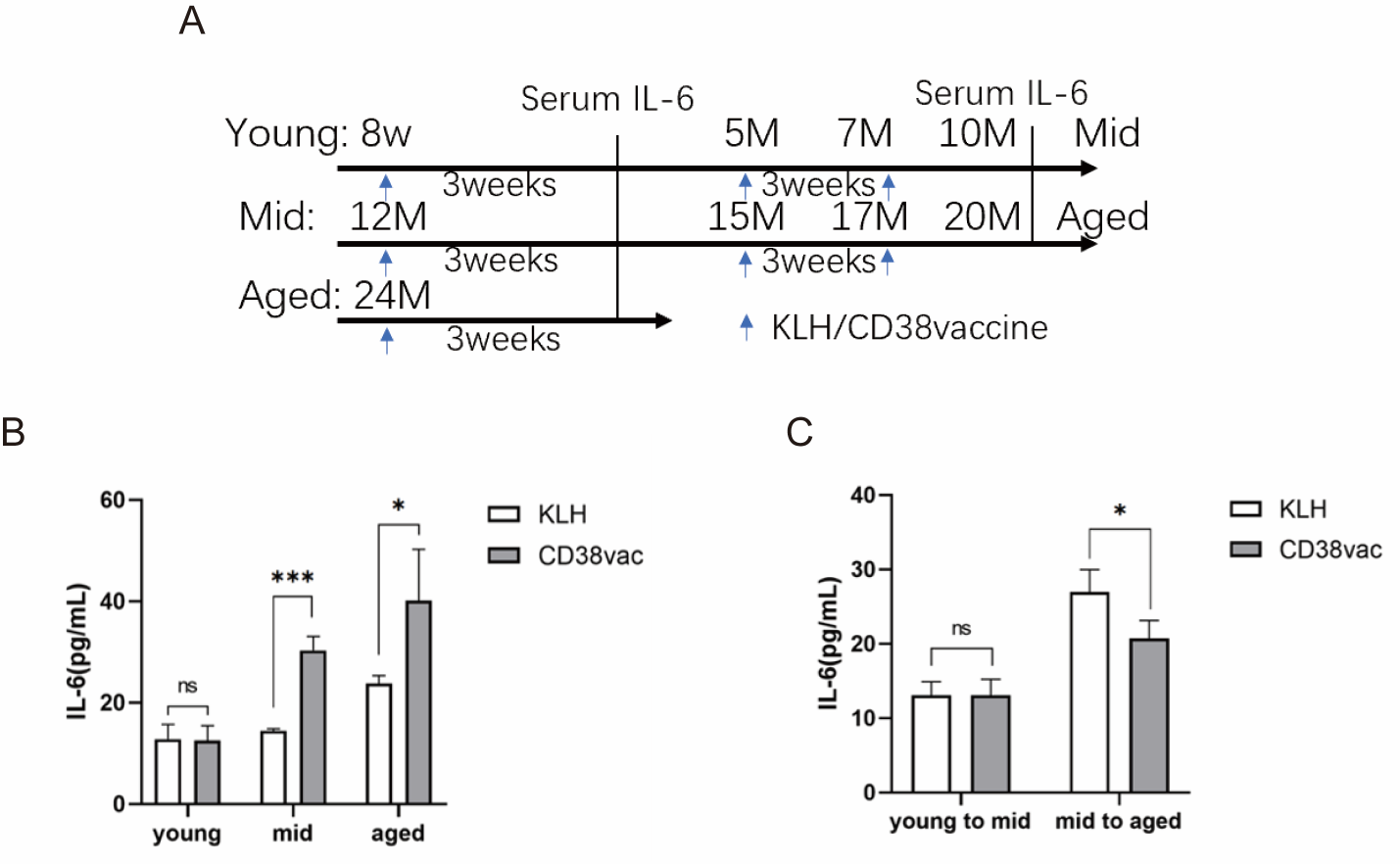


**Figure S8.** IL-6 levels in the serum of CD38-vaccinated mice at different times during vaccination. (**A**) Schematic diagrams of the vaccination and serum collection time. (**B**) Serum IL-6 levels in young (8-week-old), middle (12-month-old), and aged (24-month-old) mice three weeks after immunization with the KLH or CD38-vaccine. (**C**) Serum IL-6 levels in young and middle-aged mice after prime-boost immunization with the KLH or CD38-vaccine. Young-to-mid mice and mid-to-aged mice represent mice primarily vaccinated with the KLH or CD38 vaccine at 8 weeks old or 12 months old, following a secondary vaccination (at 5 months or 15 months) and a third vaccination (at the age of 7 months or 17 months). Sera from young-to-mid mice were collected at 10 months old, and sera from young-to-mid mice were collected at 20 months old. All data were analyzed by two-tailed Student’s *t*-test (*n* = 3/group); values represent the mean ± SD; **p* < 0.05, ****p* < 0.001, ns = not significant.


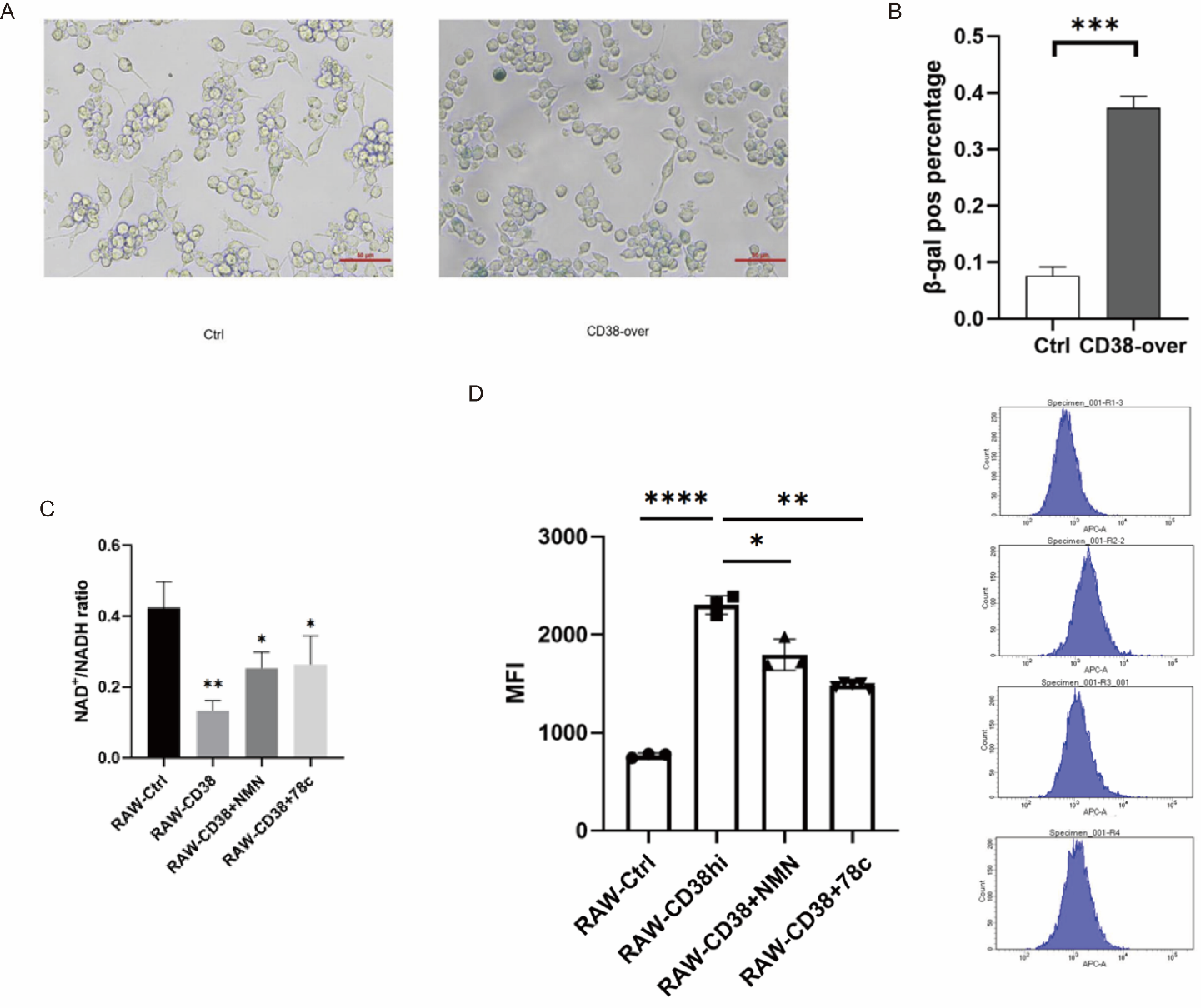


**Figure S9.** CD38 overexpression increases the proportion of senescent RAW264.7 cells. (**A**) SA-β-gal detection of RAW264.7 cells (Ctrl) and RAW264.7 cells with high CD38 expression (RAW-CD38-over). Scale bar: 50 μm. (**B**) Quantitative statistics of the proportion of senescent cells in each biological replicate (**C**) Relative NAD^+^/NADH ratio of RAW264.7 cells (RAW-Ctrl), stable CD38hi RAW264.7 cells (RAW-CD38hi), stable CD38hi RAW264.7 cells treated with 500μM NMN for 24h (RAW-CD38hi+NMN), and stable CD38hi RAW264.7 cells treated with 10μM 78c for 24h (RAW-CD38hi+78c). (**D**) Cellular ROS levels of RAW264.7 cells (RAW-Ctrl), stable CD38hi RAW264.7 cells (RAW-CD38hi), stable CD38hi RAW264.7 cells treated with 500μM NMN for 24h (RAW-CD38hi+NMN), and stable CD38hi RAW264.7 cells treated with 10μM 78c for 24h (RAW-CD38hi+78c). Data were analyzed using unpaired two-tailed t-test (B) and ANOVA followed by Tukey’s multiple comparisons test (C, D) (*n* = 3/group); values represent the mean ± SD; *p < 0.05, **p < 0.01, ***p < 0.001, ****p < 0.0001.


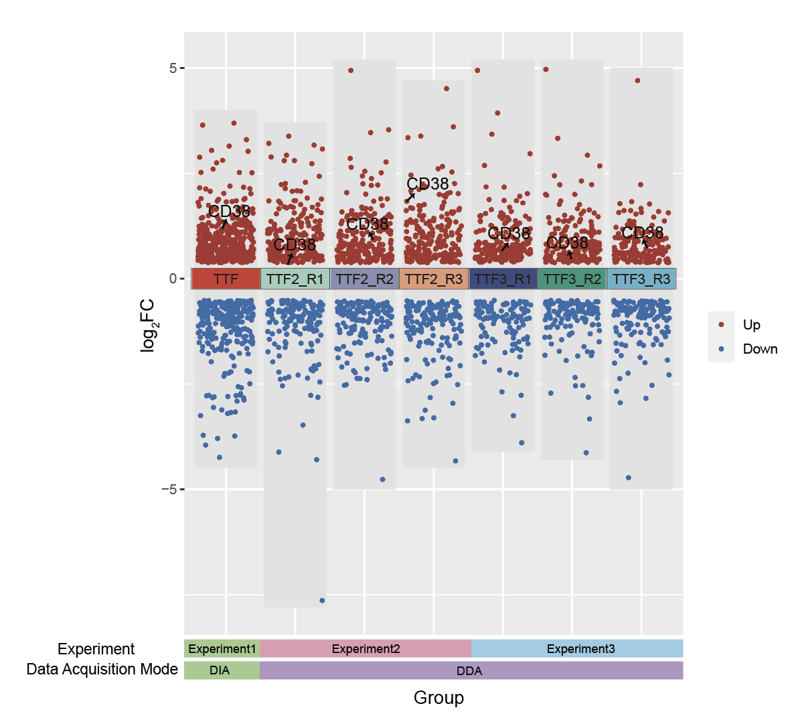


**Figure S10.** Surface omics analysis showing CD38 enrichment in aged mouse tail tip fibroblasts. The logarithm of the fold change in protein expression in mouse tail tip fibroblasts (TTFs) from aged mice compared to young mice. The data are based on three independent surface-omics experiments using different data acquisition modes (Experiment 1-DIA; Experiments 2, 3-DDA).

**Table S1.** Primers used for qPCR (**Excel online)**

**Table S2**. Predictions of MHC-I binding affinity among mouse CD38 peptides (**Excel online)**

**Table S3.** Proteomic analysis of liver tissues from CD38 and KLH mice (**Excel online)**
